# Identification and biophysical characterization of a novel domain-swapped camelid antibody specific for fentanyl

**DOI:** 10.1016/j.jbc.2024.107502

**Published:** 2024-06-28

**Authors:** Joseph P. Gallant, Dustin Hicks, Ke Shi, Nicholas H. Moeller, Brooke Hoppe, Eric W. Lake, Carly Baehr, Marco Pravetoni, Hideki Aihara, Aaron M. LeBeau

**Affiliations:** 1 Department of Pathology and Laboratory Medicine, University of Wisconsin School of Medicine and Public Health, Madison, Wisconsin, USA; 2 Department of Pharmacology, University of Minnesota Medical School, Minneapolis, Minnesota, USA; 3 Department of Biochemistry, Molecular Biology, and Biophysics, University of Minnesota, Minneapolis, Minnesota, USA; 4 Department of Psychiatry and Behavioral Sciences, University of Washington School of Medicine, Seattle, Washington, USA; 5 Center for Medication Development for Substance Use Disorders, University of Washington, Seattle, Washington, USA; 6 Department of Radiology, University of Wisconsin School of Medicine and Public Health, Madison, Wisconsin, USA; 7 Carbone Cancer Center, University of Wisconsin-Madison, Madison, Wisconsin, USA

**Keywords:** synthetic opioids, overdose, opioid addiction, antibodies, crystal structure, domain-swapped dimer

## Abstract

Opioid use disorders (OUD) and overdoses are ever-evolving public health threats that continue to grow in incidence and prevalence in the United States and abroad. Current treatments consist of opioid receptor agonists and antagonists, which are safe and effective but still suffer from some limitations. Murine and humanized monoclonal antibodies (mAb) have emerged as an alternative and complementary strategy to reverse and prevent opioid-induced respiratory depression. To explore antibody applications beyond traditional heavy-light chain mAbs, we identified and biophysically characterized a novel single-domain antibody specific for fentanyl from a camelid variable-heavy-heavy (VHH) domain phage display library. Structural data suggested that VHH binding to fentanyl was facilitated by a unique domain-swapped dimerization mechanism, which accompanied a rearrangement of complementarity-determining region loops leading to the formation of a fentanyl-binding pocket. Structure-guided mutagenesis further identified an amino acid substitution that improved the affinity and relaxed the requirement for dimerization of the VHH in fentanyl binding. Our studies demonstrate VHH engagement of an opioid and inform on how to further engineer a VHH for enhanced stability and efficacy, laying the groundwork for exploring the *in vivo* applications of VHH-based biologics against OUD and overdose.

Opioid use disorder (OUD) affects over 2.7 million people in the United States (Centers for Disease Control and Prevention, 2022). Since 2020, drug-related fatal overdoses surpassed the 100,000 mark, and over 75% were attributed to the influx of illegally synthetized opioids. Nearly two-thirds of the opioid-related overdose deaths last year were due to a single synthetic opioid, fentanyl (1, 2), which is presented either alone or more often in combination with other narcotics or stimulants. Fentanyl belongs to a class of synthetic piperidine-based opioids that act as agonists of the μ-opioid receptors (MORs) in the brain (3). When compared to opiates originating from natural products (*e.g.*, heroin or morphine), or semi-synthetic opioids like oxycodone, fentanyl is 50 to 100 times more potent (3, 4). Commonly used for pain management and as a sedative, fentanyl is fast acting and can diffuse across the blood-brain barrier (4). Due to its high potency and prolonged serum half-life (t_1/2_= 4-8h) compared to traditional opioids such as heroin, misuse of fentanyl often results in overdose that ultimately leads to severe respiratory depression and death (3, 5). Fentanyl is also found in counterfeit pills and other street drug mixtures resulting in an increased number of accidental overdoses (6, 7).

The most common pharmacological agent to reverse opioid-related overdose is the MOR antagonist naloxone. While naloxone is generally effective at reversing overdose, rapid clearance from the brain results in a short duration of action (8), relative to the half-life of fentanyl and its analogs. The transient effect and short half-life of naloxone (30–90 min) can result in renarcotization when used to treat overdose caused by longer-lasting, potent synthetic MORs agonists (8, 9, 10). Due to the perceived and real shortcomings of current naloxone formulations, the FDA has recently approved products containing higher naloxone doses as well as the longer-acting MOR antagonist nalmefene (*e.g.*, OPVEE). Current products used in medication-assisted treatment also include extended-release formulations of the MOR antagonist naltrexone (Vivitrol). To provide additional and complementary options to pharmacotherapies, pre-clinical and clinical development of immunotherapies against OUD and overdose have been actively pursued. Unlike MOR antagonist interventions, vaccines and monoclonal antibodies (mAbs) directly alter the pharmacokinetics of the target opioid by sequestering it in the serum and preventing its distribution to the MORs in the brain (11, 12). Compared to small-molecule antagonists, anti-opioid vaccines and mAbs can offer effective protection for weeks or months and confer selective activity that does not interfere with agonists and other critical medications (13). Vaccines that induce opioid specific polyclonal antibodies have demonstrated efficacy at reducing opioid biodistribution, respiratory depression, and death in preclinical rodent and non-human primate studies (14, 15, 16). A first-in-human clinical trial for a vaccine to treat OUD is currently ongoing (NCT04458545). A successful vaccine is dependent on the generation of high levels of polyclonal antibodies against the target opioid (17). This may require multiple immunizations to achieve high antibody concentrations in a subset of patients. To circumvent the variability of the immune response and to provide immediate overdose reversal against long-lived opioids, mAbs that recognize opioids including fentanyl have been developed (18, 19, 20). Recently, the human IgG1 mAb CSX-1004 received fast track designation for the prevention of overdose from fentanyl and other synthetic opioids (NCT06005402) (19). Our team is also pursuing IND-enabling studies of a humanized mAb against fentanyl (21).

Recombinant mAbs are a promising class of therapeutics to counteract the effects of opioids. All mAbs against opioids, nicotine, and other stimulants were initially identified using hybridoma technology, mammalian cell display, or direct B cell receptor sequencing from mouse, rat, and human sources (22). All of the anti-opioid mAbs reported in the literature thus far have been canonical heavy and light-chain antibodies. Single-domain antibodies represent an unexplored technology for the development of next-generation mAbs for opioids and other drug targets. Variable-heavy-heavy domains (VHHs) or nanobodies are single-domain heavy-chain only fragments found in members of the camelidae family (camels, llamas, alpacas, *etc.*) (23). VHHs have three complementarity determining regions (CDRs) for target engagement as opposed to the six CDRs (3 heavy, three light) found in human antibodies. However, the CDR3 of camelids can be over 20 amino acid residues long (24). This provides them with a unique architecture and binding interface that allows them to recognize epitopes inaccessible to canonical human and mouse antibodies (25, 26). VHHs are easy to express in large quantities because of their high solubility and require little to no humanization due to their high homology with human heavy chains (>95%), which makes VHHs ideal for translation (24, 27).

Here, we describe the identification of novel VHHs for fentanyl using a naïve VHH phage display library screened against a fentanyl hapten. Our lead VHH (JGFN4) was specific for fentanyl and displayed no cross-reactivity with the closely related synthetic opioid carfentanil. The crystal structure of JGFN4 in the presence of fentanyl revealed a homodimer with each protomer bound to one fentanyl molecule. Interestingly, the homodimer formed as a result of reciprocal swapping of the C-terminal β-strand of each protomer, mediated by an extended conformation and intermolecular interaction of CDR3. In an atypical fashion, CDR3 did not form key interactions with fentanyl; rather, binding was through CDR1 and CDR2. Through rational mutagenesis and additional crystallographic analysis, we identified a mutation that allowed monomeric JGFN4 to bind fentanyl, abrogating the need for domain swapping. Biolayer interferometry (BLI) and differential scanning fluorimetry (DSF) determined that mutant versions of JGFN4 had increased affinity for fentanyl compared to wild-type JGFN4 (JGFN4 WT). Our study documents an important proof-of-concept showing that single-domain antibodies can engage synthetic opioids, thus warranting further investigation of these domains as a novel class of therapeutics for treating OUD.

## Results

### Identification of a VHH specific for fentanyl

**Figure 1:**
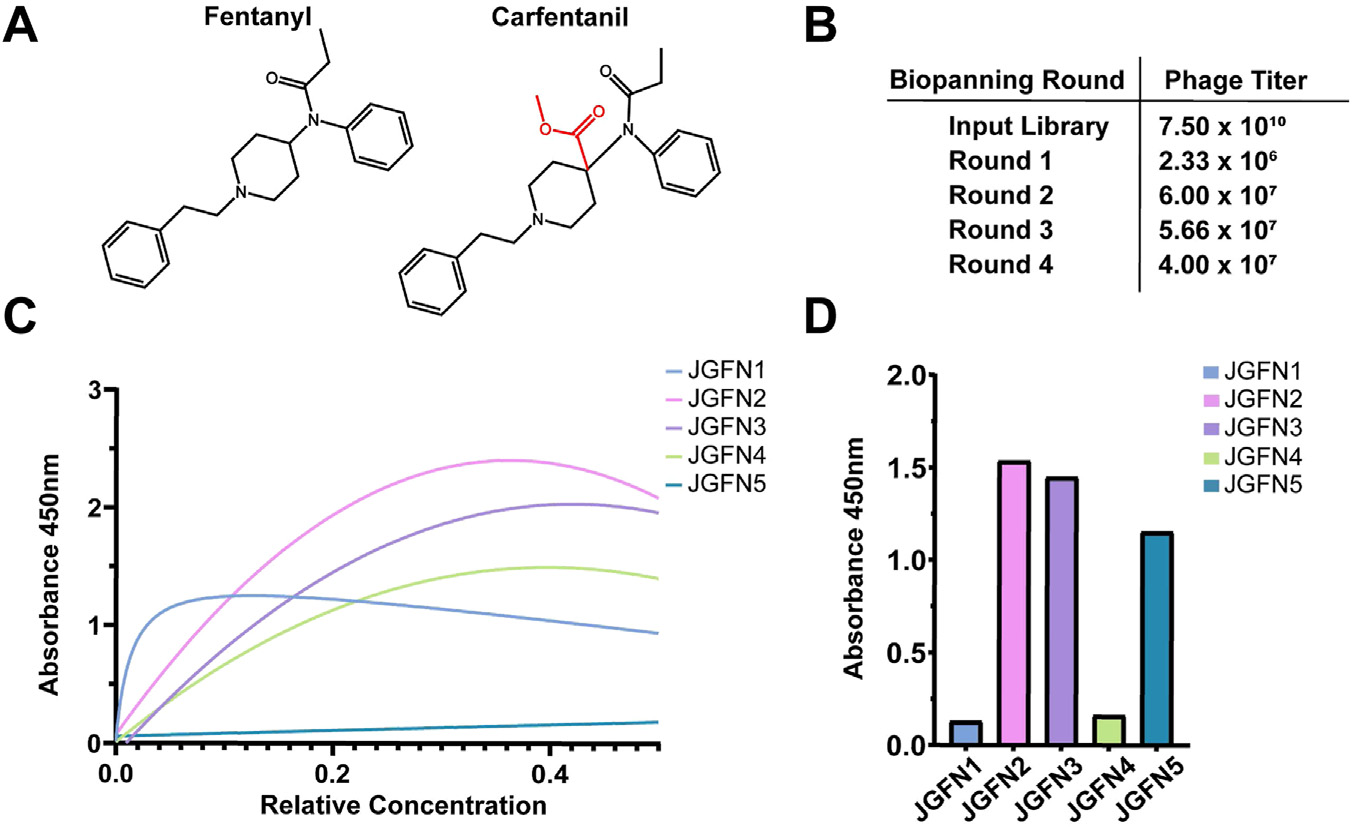
**Naive V_HH_ biopanning campaign identifies lead anti-fentanyl V_HH_.***A*, structures of fentanyl (CAS: 437-38-7) and carfentanil (CAS: 59708-52-0) with structural differences highlighted in *red*. *B*, camelid phage display library used for biopanning campaign and the resulting phage titers following each round. *C*, dilution ELISA of the five lead anti-fentanyl camelids against an immobilized fentanyl hapten conjugated to BSA (F_3_-BSA) (28) showing saturable binding profiles. *D*, the highest concentration from the dilution ELISA in (*C*) was evaluated in a single concentration ELISA against an immobilized carfentanil hapten (F_11_) (28).

To identify a fentanyl-specific VHH, we screened our in-house naive VHH phage display library against a biotinylated fentanyl hapten (previously described as F_3_) (28, 29) immobilized on magnetic beads (Figs. 1 and S1). The initial round of biopanning resulted in a large reduction of phage particles as compared to the starting library, indicative of a successful reduction of non-binding VHH variants. The second round of biopanning yielded an increased phage titer, suggesting there was an enrichment of opioid-binding antibodies from Round 1. The last two rounds of biopanning conserved this phage titer and no indication of a diversity collapse was found in the final rounds of biopanning (Fig. 1*B*). The supernatant of individual VHH clones induced in monoculture following Rounds 3 and 4 (totaling 672) were tested by ELISA for binding to fentanyl haptens. Clones that demonstrated a high ELISA signal (>0.75) were sent for sequencing. Sequencing identified a total of five unique camelid VHH sequences, denoted as JGFN 1 to 5 (Fig. S2), that had varying affinity and selectivity for fentanyl and carfentanil. Carfentanil is a highly potent synthetic opioid that differs from fentanyl by the addition of a methyl ester to the piperidine ring (Fig. 1*A*). Evaluation of these five lead VHHs for cross-reactivity against the screened fentanyl hapten F_3_ and a homologous carfentanil hapten (F_11,_ as previously described) (30) revealed that JGFN one and JGFN four were fentanyl-specific while JGFN two and JGFN three showed binding to both fentanyl and carfentanil. Interestingly, JGFN five showed very weak binding to fentanyl when compared to carfentanil (Fig. 1, *A*, *C*, and *D*).

### JGFN4 forms a domain-swapped dimer to bind fentanyl

**Figure 2:**
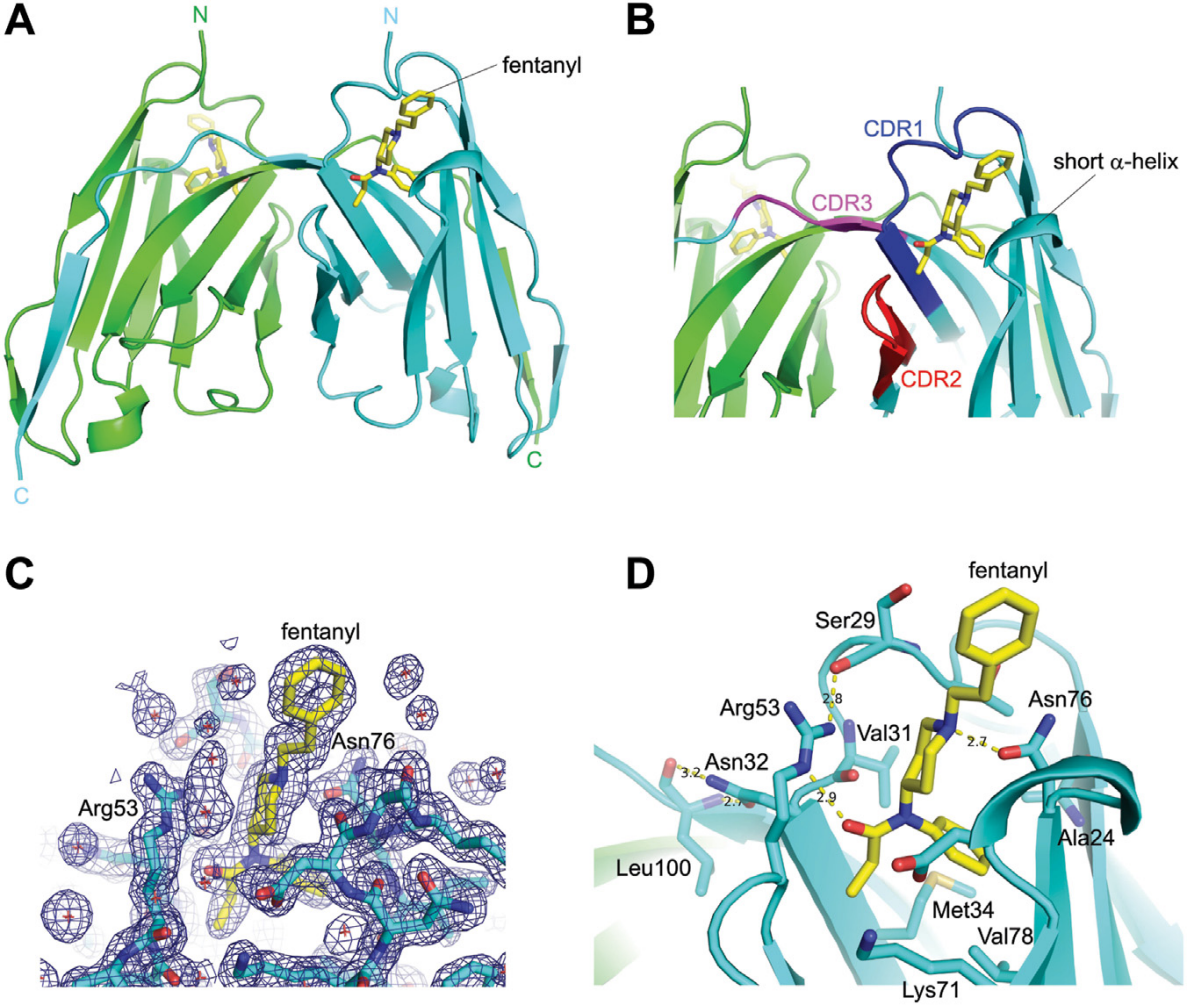
**JGFN4 binds fentanyl as a domain-swapped dimer.***A*, overall structure of the domain-swapped JGFN4 dimer bound to fentanyl. *B*, the CDR 1 to 3 loops of JGFN4 in the domain-swapped ‘*trans*’ configuration. *C*, 2Fo-Fc electron density contoured at 1.0 sigma. *D*, a close-up view of the fentanyl-binding pocket, with the side chains of key amino acid residues shown. *Yellow dashed lines* indicate hydrogen bonds, with distances shown in angstroms.

We next sought to determine the crystal structure of JGFN4 with and without fentanyl to understand how the VHH recognizes fentanyl. JGFN4 expressed in *E. coli* was purified as a mixture of monomers and non-covalent homodimers, separable by size-exclusion chromatography (Figs. S3 and S4). Both the monomeric and non-covalent dimeric forms of JGFN4 were subjected to crystallization screening and yielded crystals in the presence of fentanyl. The structures were determined by molecular replacement phasing and refined to 1.68 and 1.55-Å resolution for crystals obtained with the monomeric and dimeric species, respectively (Table 1). Unexpectedly, both crystal structures showed a domain-swapped homodimer of JGFN4, with each protomer bound to one fentanyl molecule (Fig. 2*A*). Fentanyl was surrounded by CDR1, CDR2, and a juxtaposed loop between CDR2 and CDR3, which formed a turn of a helix (Fig. 2*B*). The CDR3, which typically forms a long loop responsible for making key interactions with the antigen by VHHs (31), instead crossed over to the other protomer (*trans* conformation) to mediate a reciprocal swapping of the C-terminal β-strand of JGFN4.

**Table 1:** Crystallization data collection and refinement statistics

Structure	WT dimer-fentanyl	WT monomer-APO	Preformed WT dimer-fentanyl	N76D monomer-fentanyl	N76D dimer-fentanyl
PDB ID	8V9W	8V9Y	8V9X	8V9Z	8VA0
Data collection					
Space group	*P*2_1_2_1_2	*C*2	*C*222_1_	*C*2	*C*222_1_
Unit cell dimensions					
*a, b, c* (Å)	85.43, 97.14, 57.58	91.24, 26.86, 36.96	44.79, 109.07, 50.20	91.10, 26.36, 37.46	57.31, 83.85, 96.61
α, β, γ (º)	90, 90, 90	90, 106.99, 90	90, 90, 90	90, 107.19, 90	90, 90, 90
Resolution (Å)	47.8–1.68 (1.74–1.68)	43.6–1.76 (1.81–1.76)	36.9–1.55 (1.58–1.55)	43.51–1.60 (1.63–1.60)	47.3–1.85 (1.89–1.85)
*R*_sym_ or *R*_merge_	0.0717 (1.04)	0.061 (0.279)	0.099 (1.63)	0.094 (1.94)	0.050 (0.839)
*I/σI*	9.80 (1.26)	10.4 (2.5)	8.14 (0.78)	4.80 (0.60)	19.41 (2.30)
Completeness (%)	97.43 (97.74)	91.40 (59.7)	97.41 (91.89)	97.7 (97.9)	99.20 (99.25)
Redundancy	3.7 (3.7)	3.6 (2.2)	4.3 (3.3)	2.7 (2.7)	7.6 (7.6)
CC_1/2_	0.996 (0.677)	0.998 (0.901)	0.996 (0.436)	0.965 (0.450)	1.000 (0.875)
Refinement					
Resolution (Å)	47.8–1.68 (1.74–1.68)	35.4–1.76 (1.82–1.76)	36.9–1.55 (1.61–1.55)	35.8–1.6 (1.66–1.60)	47.3–1.85 (1.92–1.85)
No. Reflections	53,990 (5330)	7971 (532)	17,815 (1643)	11,139 (1059)	20,120 (1976)
*R*_work_/*R*_free_	0.200/0.239	0.191/0.232	0.187/0.218	0.234/0.267	0.203/0.240
No. atoms	3921	918	1016	908	1867
Protein	3406	857	852	845	1695
Ligand/ion	104	0	29	25	68
Water	411	61	135	38	104
*B*-factor	34.97	20.92	28.70	39.07	44.83
Protein	33.93	20.73	27.66	38.99	44.83
Ligand/ion	40.09	n.a.	23.78	42.26	44.73
Water	42.34	23.56	36.36	38.86	45.48
R.m.s. deviations					
Bond lengths (Å)	0.015	0.003	0.003	0.002	0.008
Bond angles (º)	1.30	0.60	0.56	0.495	0.94

Statistics for the highest-resolution shell are shown in parentheses.

### The JGFN4 binding pocket relies on hydrophobic contacts, bulky side chains, and hydrogen bonding to bind to fentanyl

Analysis of the X-ray crystal structure elucidated the specific interactions between the binding pocket and fentanyl. The N-phenylpropanamide moiety of fentanyl is inserted into a deep pocket formed between the two layers of β-sheets that form the core of the VHH. Hydrophobic contacts are made with A24, M34, K71, V78, and R53 side chains and van der Waals contacts with the main chain atoms of V31 and N32 (Fig. 2, *C* and *D*). In CDR2, R53 also donates a hydrogen bond to the carbonyl oxygen atom of fentanyl and to the S29 carbonyl group from CDR1 to help shape the pocket. Hydrogen bonding is also observed between N76 and the tertiary amine nitrogen atom of the piperidine moiety on fentanyl. The piperidine ring is also stabilized by extensive van der Waals contacts with protein residues from CDR1 and the short helix between CDR2 and CDR3 mentioned above. The phenyl ethyl moiety N-linked to the piperidine was pointed away from the protein and exposed to the solvent, where its conformation was crystal form-dependent.

**Figure 3:**
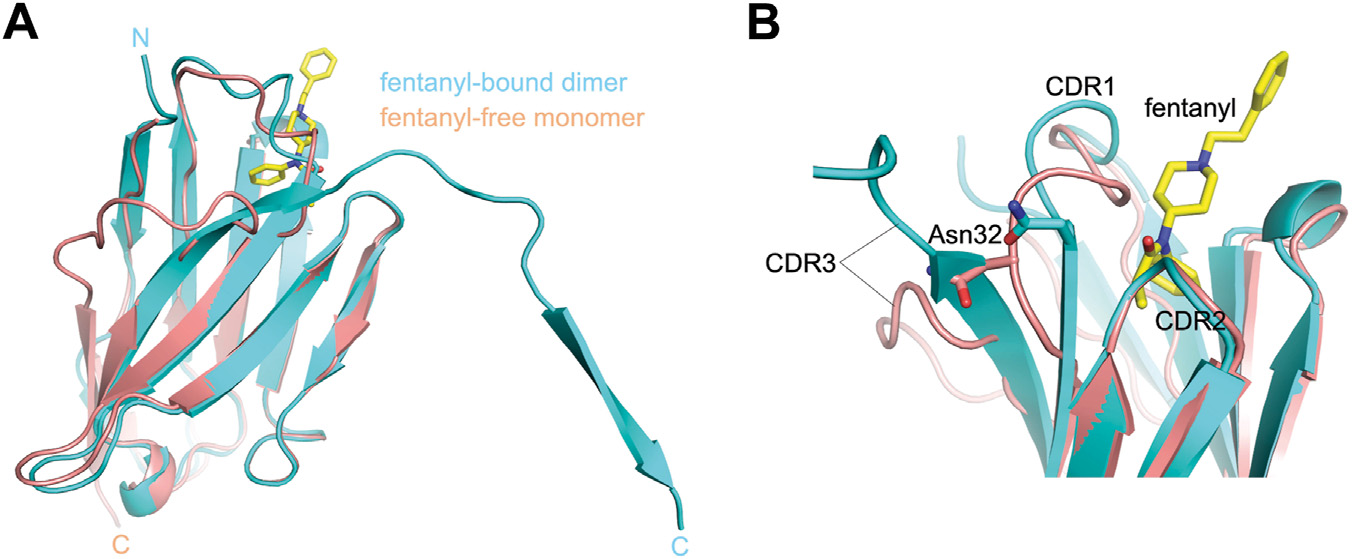
**Alternative (*cisversustrans*) conformations of JGFN4.***A*, superposition between a fentanyl-free JGFN4 monomer (*beige*) and a fentanyl-bound JGFN4 protomer form with the domain-swapped homodimer (*cyan*), highlighting distinct configurations of the C-terminal β-strand. *B*, a close-up view of the superposition in A, showing different conformations of CDR3 and the neighboring CDR1 including N32.

We obtained crystals of isolated monomeric JGFN4 in the absence of fentanyl and determined the structure at a 1.76-Å resolution (Fig. S5). The structure shows monomeric JGFN4 without domain swapping—the CDR3 forms a turn in this structure (*cis* conformation), allowing the C-terminal β-strand to complete the VHH fold intramolecularly (Fig. 3). The CDR1 loop partially collapses into the unoccupied fentanyl-binding pocket. Patchy electron density and high B-factors in the refined model suggest that the CDR1 loop is highly flexible in the absence of fentanyl. These observations suggest that JGFN4 preferentially binds fentanyl in the domain-swapped dimer and that crystallization in the presence of fentanyl likely promoted VHH dimerization. A superposition of the fentanyl-free JGFN4 monomer with the fentanyl-bound JGFN4 dimer highlights distinct conformations of CDR3 and neighboring structural elements (Fig. 3*A*). Of particular interest is N32 from CDR1, whose side chain undergoes a ∼4.5 Å shift upon the transition of CDR3 from the *cis* to *trans* conformations (Fig. 3*B*). In the *trans* conformation, L100 from the straightened CDR3 formed bidentate hydrogen bonds with the side chain of N32, which accompanies a ∼3 Å shift of the Cα position to shape the fentanyl-binding pocket.

### Mutations increase the affinity for fentanyl in dimerized JGFN4

**Figure 4:**
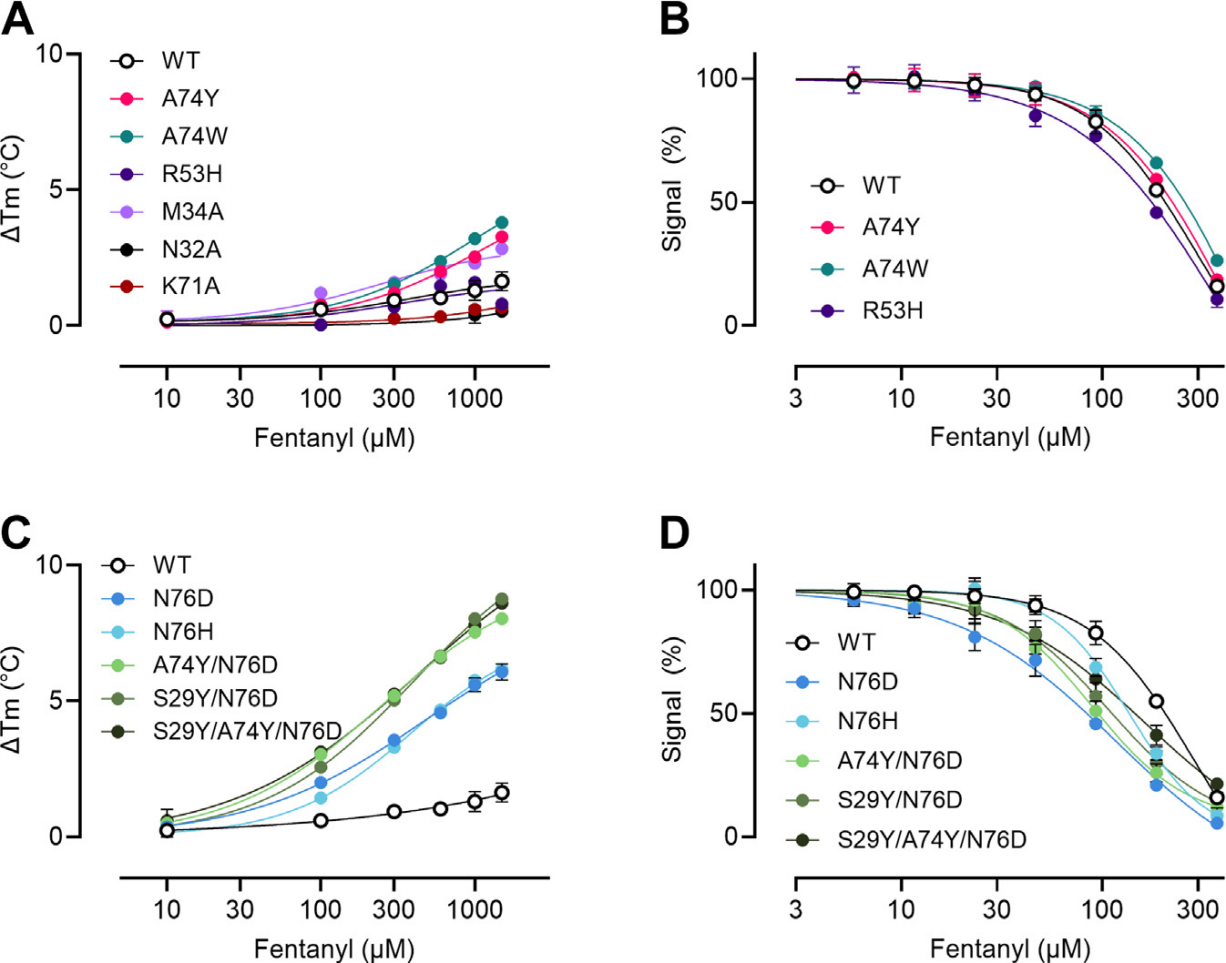
**DSF and competitive ELISA of WT and mutant JGFN4 VHH dimers in the presence of free fentanyl.***A* and *C*, fentanyl-dependent increase in melting temperature determined by DSF showing stabilization of VHH dimer by binding to fentanyl. Data are mean ± SD of three replicates per sample. *B* and *D*, competitive ELISA showing inhibition of VHH binding to plate coated with fentanyl hapten by free fentanyl. Y-axis normalized to max absorbance signal. Data are mean ± SD of of four replicates. M34A, N32A, and K71A mutants were not included in competitive ELISA due to poor binding to fentanyl hapten. For clarity, VHH samples in (*A*–*D*) are separated by “low affinity” and “high affinity”, respectively.

Based on the crystallographic studies indicating that JGFN4 bound fentanyl as a dimer, we expressed JGFN4 as a homodimer by fusing two VHH domains of JGFN4 with a (Gly_4_Ser)_4_ linker to force dimerization (Fig. S6). Rational mutagenesis using structural data as guidance was next performed to improve binding. Measurements of affinity were performed using orthogonal methods of biolayer interferometry (BLI), which is a kinetic method of affinity determination using immobilized F_1_ hapten, and competitive ELISA and differential scanning fluorimetry (DSF), which use free fentanyl (21). BLI is likely to overestimate the affinity of dimeric proteins due to the reduced off-rate when both binding sites are bound to the sensor (increased avidity), which does not accurately reflect the behavior of free fentanyl binding in solution. To verify the binding of JGFN4 WT and the mutants to free drug, competitive ELISA and DSF were employed (Table 2, Figs. 4, S8–S11, and Tables S1–S7).

**Table 2:** F_1_ fentanyl hapten (BLI) and free fentanyl (Competitive ELISA and DSF) binding data for mammalian expressed and purified WT and mutant JGFN4 recombinant homodimers

JGFN4 dimer mutant ID	BLI K_D_ (M)		Comp. ELISA IC_50_ (M)		DSF K_D_^a^ (M)	
WT	1.07E-08	±8.10E-10	2.26E-05	±5.11E-06	1.10E-03	±8.91E-05
A74Y	1.20E-08	±6.51E-11	2.48E-05	±2.93E-06	4.35E-04	±3.01E-05
A74W	1.63E-08	±1.13E-09	3.61E-05	±1.14E-05	3.23E-04	±9.33E-06
R53H	No Binding		1.78E-05	±2.49E-06	8.67E-04	±1.54E-04
N76D	8.46E-09	±6.54E-10	*4.43E-06*	±1.78E-07	8.65E-05	±1.03E-06
N76H	1.36E-08	±1.46E-10	7.95E-06	±4.30E-07	8.01E-05	±3.27E-06
A74Y/N76D	*5.80E-09*	±2.05E-09	*4.14E-06*	±3.90E-07	4.26E-05	±1.54E-06
S29Y/N76D	*5.45E-09*	±4.38E-10	5.35E-06	±5.46E-07	6.46E-06	±2.00E-07
S29Y/A74Y/N76D	*5.43E-09*	±5.01E-10	9.58E-06	±2.50E-06	*5.60E-07*	±4.00E-08
M34A	No Binding		No Binding		8.74E-04	±1.39E-05
K71A	No Binding		No Binding		7.36E-04	±9.60E-03
N32A	No Binding		No Binding		3.80E-02	±3.73E-02

Highest affinity binder(s) for each measure denoted by *italics*. BLI error reported as 1 standard deviation. Competitive ELISA and DSF error reported as ± SEM of four and three replicates per sample, respectively. See Supporting Information for full data sets.

a As determined at 600 uM free fentanyl concentration.

The binding affinity of dimerized JGFN4 WT for immobilized fentanyl hapten F_1_ was determined to be 10.7 nM by biolayer interferometry (BLI) (avidity-influenced), 22.6 μM by competitive ELISA, and 1.1 mM by DSF (Table 2) (avidity-independent). Data suggest that hydrogen bonding between N76 and the piperidine moiety of fentanyl is a key interaction for VHH engagement. Previously, we identified a similar interaction with our murine-derived anti-fentanyl antibody HY6-F9 where an Asn residue formed a hydrogen bond with the piperidine moiety (20). Therefore, we generated a point mutant N76D and N76H of JGFN4 in an attempt to strengthen its polar interaction with the piperidine moiety. Further, A74 was mutated to Tyr or Trp to promote possible π-stacking with the phenethyl group of fentanyl. While N76H did not show an affinity improvement by BLI, JGFN4 N76D showed a modest increase in affinity. However, by competitive ELISA and DSF, both showed improved affinity relative to WT. The A74Y and A74W mutations did not result in a major improvement in affinity by any measure (Table 2). When combined, the A74Y/N76D double mutants displayed a BLI K_D_ of 5.8 nM, and a slight improvement over the N76D mutant alone by competitive ELISA and DSF. An additional mutation (S29Y) expected to further increase π-stacking interactions with the phenylethyl group was investigated with N76D (S29Y/N76D) or in combination with N76D and A74Y (S29Y/A74Y/N76D). While these mutants did not show a major affinity improvement by BLI or competitive ELISA, the presence of Tyr residues in proximity to the phenethyl group of fentanyl appear to result in additional thermal stabilization of the fentanyl:VHH complex, as S29Y/N76D and S29Y/A74Y/N76D resulted in a ∼170- and ∼2000-fold improvement in K_D_ against free fentanyl by DSF. Double mutant N76Y/T28D was also assessed for potentially optimized interactions with the phenylethyl group (N76Y) and coordination with the tertiary amine (T28D); however, fentanyl hapten binding by BLI was ablated, highlighting the importance of an Asn or Asp at position 76 for interacting with the tertiary amine. We next made mutations in residues that formed key interactions with N-phenylpropanamide moiety of fentanyl that would either decrease or inhibit binding. JGFN4 WT mutants M34A, K71A, N32A, and R53H resulted in ablated or greatly reduced binding to F1 fentanyl hapten underscoring the importance residues for engagement. The reduced binding of R53H indicated that while hydrogen bonding to the carbonyl oxygen of fentanyl may have been maintained, the interaction between R53 and S29 is critical for fentanyl binding and could not be mimicked by the substituted histidine.

Overall, structure-guided mutations resulted in improved binding to fentanyl. JGFN4 WT displayed the lowest binding to fentanyl by competitive ELISA and DSF with an IC_50_ of 22.6 ± 5.11 μM and K_D_ of 1.1 ± 0.089 mM, respectively. By BLI, N76D mutants containing one or both S29Y and A74Y mutations displayed the highest affinity for fentanyl hapten with a K_D_ of 5.43 to 5.80 nM. By competitive ELISA, JGFN4 N76D and JGFN4 A74Y/N76D displayed the highest affinity, with an IC_50_ of 4.14 to 4.43 μM. By DSF, JGFN4 S29Y/A74Y/N76D displayed the highest affinity, with a K_D_ of 0.560 uM. While DSF is suitable for a comparison of K_D_ between highly similar proteins (*i.e.* point mutants compared to a WT control), there are limitations to this technique as K_D_ is determined at physiologically irrelevant temperatures, and results from this method should not be directly compared to results from other methods. With three orthogonal measures of binding affinity, we were able to thoroughly assess the impact of several different mutations to a novel VHH that binds fentanyl.

### N76D mutation allows JGFN4 monomers to bind fentanyl

**Figure 5:**
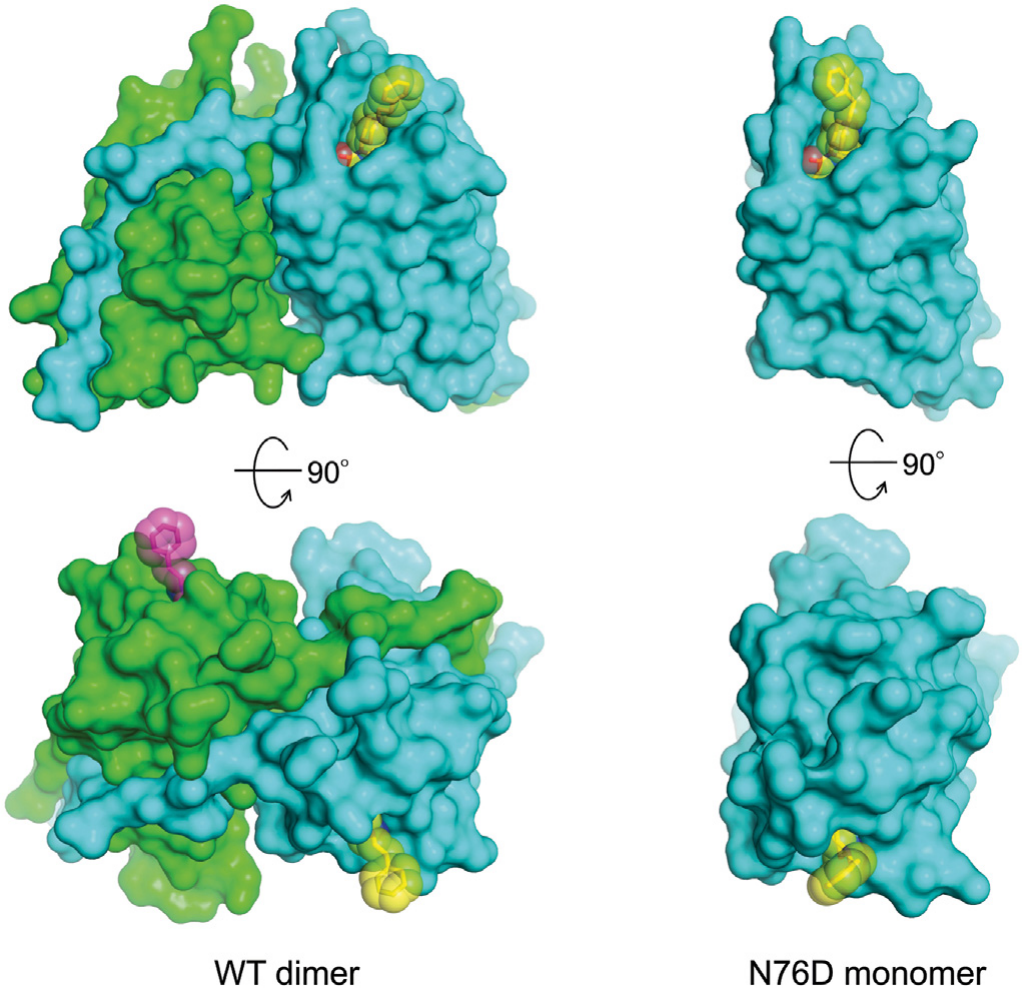
**Fentanyl binding with or without domain swapping.** Protein surfaces of the domain-swapped JGFN4 WT dimer bound to fentanyl (*left*) and the JGFN4 N76D monomer bound to fentanyl (*right*), viewed from two orthogonal orientations. The fentanyl molecules are shown as sticks with transparent spheres indicating the van der Waals radii. The two fentanyl molecules bound to the JGFN4 dimer are colored *yellow* and *magenta*.

**Figure 6:**
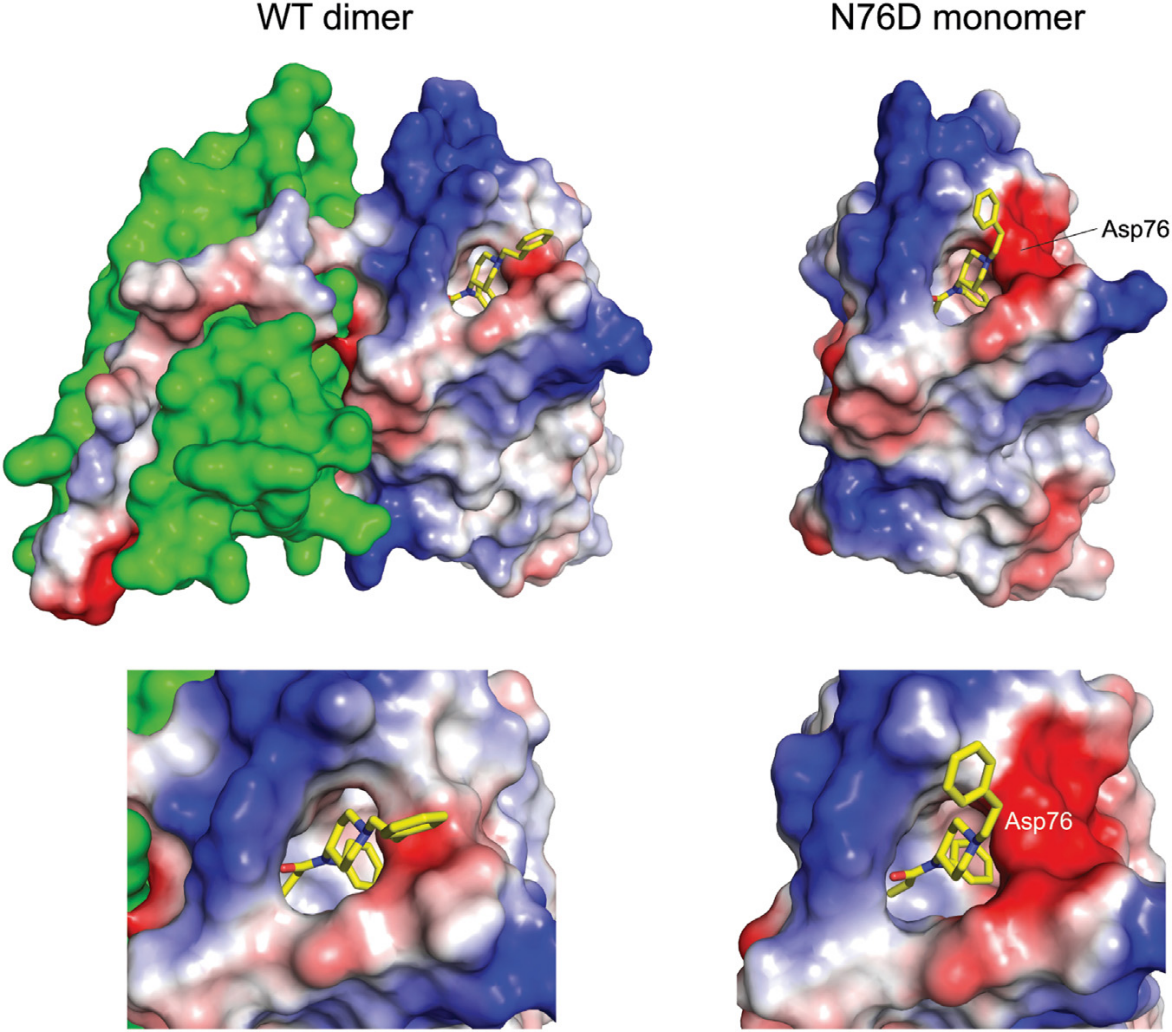
**The fentanyl-binding pocket in JGFN4 WT dimer *vs*. N76D monomer.** Molecular surfaces around the fentanyl-binding pocket of the fentanyl-bound JGFN4 WT dimer (*left*) and fentanyl-bound JGFN4 N76D monomer (*right*) with enhanced views for both below. Electrostatic surface potential (*blue*: positive, *red*: negative) is shown for one of the protomers in the JGFN4 WT and the N76D monomer. The position of the substituted D76 residue in N76D is indicated. Note that the pocket is tighter in the dimeric JGFN4 complex, whereas it is flanked by a stronger negative charge in the N76D complex.

**Figure 7:**
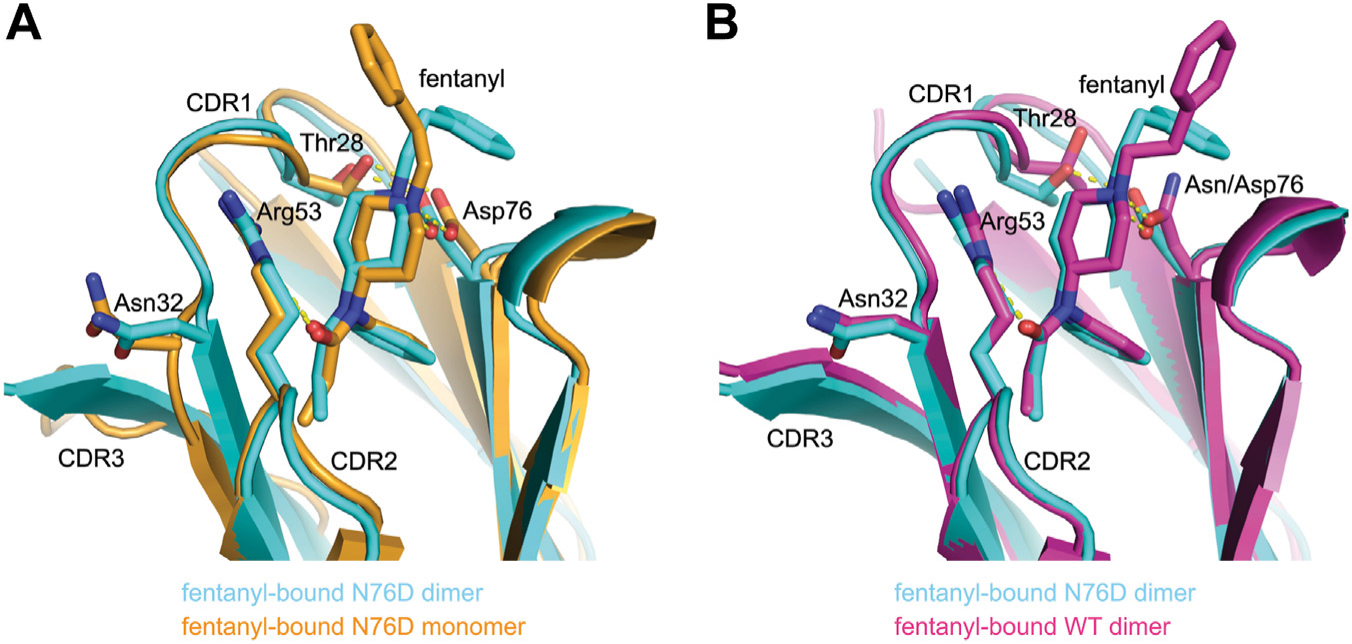
**JGFN4 N76D can bind fentanyl either as monomer or dimer.***A*, A comparison between the fentanyl-bound JGFN4 N76D monomer (*orang*e) and a protomer of fentanyl-bound JGFN4 N76D within the domain-swapped homodimer (*cyan*). Note the distinct positioning of N32. *B*, a comparison between the fentanyl-bound JGFN4 WT (*magenta*) and N76D (*cyan*), both from domain-swapped homodimers. Note that a hydrogen bond between D76 and the T28 side chain brings T28 closer to fentanyl.

We next decided to investigate the structure and detailed interactions of the N76D mutation. As observed for JGFN4 WT, we obtained monomeric and dimeric forms of JGFN4 N76D when it was expressed in *E. coli*. Both species yielded crystals in the presence of fentanyl, and the structures were determined at 1.60 and 1.85-Å resolution. As expected, crystals obtained with the dimeric form showed a domain-swapped dimer with the *trans* conformation of CDR3 and fentanyl bound to each protomer. In contrast to our predictions, however, the structure for the crystals grown with monomeric JGFN4 N76D was found to be a monomer with bound fentanyl (Fig. 5). Thus, it appears that the N76D mutation alleviates the need for JGFN4 dimerization *via* domain swapping in fentanyl binding. A comparison of the electrostatic surface potential for JGFN4 WT and JGFN4 N76D bound to fentanyl showed that the binding pocket of JGFN4 WT was narrower and less negatively charged compared to the strong negative charge in JGFN4 N76D (Fig. 6). It was found that the substituted D76 side chain forms a hydrogen bond not only with the tertiary amine nitrogen of the piperidine moiety but also with the side chain of T28 from CDR1 (Fig. 7*A*). N76 and T28 are ∼1.0 Å farther apart in the wild-type structure. This shift also brings the T28 side chain closer by ∼1.0 Å to the piperidine ring of fentanyl. Given the flexibility of CDR1 as observed in the fentanyl-free structure, stabilization of CDR1 in the fentanyl-bound conformation and an enhanced van der Waals contact elicited by the N76D mutation likely account for its improved affinity for fentanyl.

While a structural comparison between the fentanyl-bound JGFN4 WT dimer and fentanyl-free JGFN4 WT monomer was instructive, having the monomeric and dimeric JGFN4 N76D structures both in complex with fentanyl allows for a more direct comparison to understand how the JGFN4 dimerization benefits fentanyl binding. A superposition of the two structures shows that the N-phenylpropanamide moiety of fentanyl is more tightly surrounded by protein residues in the dimer (Fig. 7*B*). N32 is positioned closer to fentanyl due to its interaction with L100 in the extended *trans* conformation (*e.g.*, N32 Cα to the carbonyl oxygen atom of fentanyl is 1.2 Å closer) in the dimer. We reason that the flexibility of glycine residues in the unique CDR3 sequence of JGFN4 (^98^GVLG^101^) promotes switching to the *trans* conformation, which helps to shape a tighter binding pocket for fentanyl through its interaction with CDR1 residues including N32.

## Discussion

The widespread availability of fentanyl and its analogs has resulted in an increased number of fatal opioid overdoses in recent years. Given the unique properties of fentanyl and other synthetic opioids, the current therapies for OUD and the reversal of overdose are insufficient to counter the growing trend of opioid-related deaths. Recombinant mAbs that act by binding opioids and altering their biodistribution to the brain and other organs, rather than direct antagonism with the MORs, have the potential to ameliorate the effects of fentanyl and other synthetic opioids. Unlike anti-opioid vaccines that require weeks and several series of immunizations to generate enough antibodies to counteract fentanyl, mAbs are fast-acting and can be administered as intravenous infusions or as a single injection. These dosing properties allow mAbs to be used as prophylactics and therapies for both OUD and overdose. While several anti-opioid antibodies currently exist, they are all based on canonical heavy and light chain antibodies (18, 19, 20, 32, 33). Alternative antibody scaffolds that deviate from heavy and light chain antibodies have never been explored as countermeasures for fentanyl or other opioids. In this study, we detail the discovery of a first-in-class single-domain VHH fragment that can selectively bind fentanyl. To our knowledge, this is the first report of a single-domain antibody that specifically recognizes a synthetic opioid. Our lead VHH for fentanyl, JGFN4, was identified by phage display using a high-diversity camelid antibody library that we made from the B cells of nearly a dozen llamas and alpacas (26). Compared to traditional hybridoma technology, phage display allows for the identification of antibody clones in a short amount of time (less than a month). While the library we used in this study was naive, it is possible to develop biased libraries against different classes of synthetic opioids through the direct immunization of camelids with opioid haptens. This further underscores the utility of phage display in the discovery of new binding proteins for synthetic opioids.

Using X-ray crystallography, we found that JGFN4 WT dimerized and bound fentanyl through an unusual domain-swapping mechanism. In domain swapping, proteins dimerize or oligomerize by forming bonds between identical domains on an individual monomer (34, 35). Out of the thousands of protein structures known, only a few dozen are known to create high-order structures through domain swapping (36). A common hallmark of these proteins is that swapping occurs with domains that are located at either the N or C terminus of the molecule (34). In the case of JGFN4, domain swapping occurred *via* CDR3 at the C terminus of the VHH. A similar domain-swapping motif through CDR3 has been reported for another camelid domain, VHH-R9 (37). VHH-R9 was identified from the direct immunization of a llama with a red dye-conjugated hapten. The CDR3 and the C terminal β-sheet of VHH-R9 swapped between symmetry-related molecules to form a dimer. The dimer of VHH-R9 was induced by an N-terminal truncation which destabilized the VHH domain and allowed for domain swapping during crystallization. VHH-R9 also had the shortest CDR3 sequence of any reported VHH consisting of only four amino acids. Curiously, out of the five anti-fentanyl VHHs identified by our phage display screen, JGFN4 had the shortest CDR3 consisting of only seven amino acids which was half the size of the average CDR3 length of our other four VHHs. Typically, the average size of the CDR3 for a VHH is approximately 17 amino acids (38, 39). For JGFN4, domain swapping resulted from a combination of a short CDR3 and the unique interactions it made with surrounding residues including the N32 side chain in CDR1.

The key interactions that determine the binding mode and specificity of canonical heavy and light chain antibodies typically occur through the heavy chain CDR3 (40, 41). This is also observed with single-domain antibodies where the flexible and diverse CDR3 is responsible for a majority of binding interactions (26, 42). With JGFN4, however, the binding pocket and stabilizing interactions were formed by CDR1 and CDR2. The recognition of the fentanyl by JGFN4 was driven by hydrophobic interactions between the bottom of the binding pocket with the N-phenylpropanimide moiety and hydrogen bonding with a tertiary amine in the piperidine ring. JGFN4 was specific for fentanyl due to the architecture of the binding pocket which likely resulted in a steric clash with the methyl ester group of carfentanil. In JGFN4, no fentanyl binding occurred through the solvent-exposed phenethyl group. Mutations were made to JGFN4 that enhanced fentanyl binding. In particular, the N76D mutation resulted in increased affinity due to greater hydrogen bonding with the protonated tertiary amine of the piperidine moiety paired with a more optimally shaped binding pocket, and mutations that attempted to coordinate the phenethyl group of fentanyl appear to have modestly improved binding and the overall stability of the VHH:fentanyl complex. JGFN4 N76D was able to still form a domain-swapped dimer and we were able to crystalize it in both monomeric and dimeric forms in complex with fentanyl. Unlike the wild type, JGFN4 N76D was able to bind fentanyl as a monomer. Future structure-based engineering of JGFN4 to improve its affinity for fentanyl should take these factors into account, in particular, the likely benefits of enhanced contact with the phenylethyl group.

The binding mode of JGFN4 for fentanyl shares many similarities with other fentanyl-binding mAbs in the literature. This can be attributed to the small number of interactions that can occur with fentanyl due to its limited molecular space. Recently, we published the structure of our mAb HY6-F9 complexed with fentanyl (20). Here, the heavy chain CDR3 loops move into place following fentanyl binding *via* an induced fit mechanism. A hydrogen bond with the tertiary amine of the piperidine ring is then formed and an Asn residue in the CDR3 secures fentanyl in place through a clamp-like action. Hydrogen bonding with the tertiary amine of the piperidine ring appears to be a conserved protonated amine opioid-drug binding mechanism among the documented mAbs. Two recently reported mAbs, FenAb609 and FenAb208, were found to bind fentanyl *via* a deep pocket formed by the heavy and light chains (43). The N-phenylpropanimide moiety of fentanyl recognized the bottom of the pocket with the phenethyl moiety pointing away from the pocket much like JGFN4. The antibody C10-S66K was discovered through immunization with a hapten that had a linker to fentanyl in the opposite orientation of our F_3_ hapten that was used to identify JGFN4 (44). As a result, C10-S66K bound fentanyl in a reverse orientation where interactions occurred predominantly with the phenethyl and piperidine groups (19). This binding orientation allowed the mAb to recognize several synthetic opioids in addition to fentanyl.

Single-domain antibodies are a promising class of therapeutics for OUD given their unique properties when compared to canonical antibodies. Their binding fragments are typically 12-15 kDa in molecular weight which is a fraction of the size of a human or mouse fragment-antigen binding domain (50 kDa) (38). The decreased molecular weight can allow for alternative routes of administration including intranasal which would be advantageous for overdose victims and first responders (45, 46). Additionally, there are several reports documenting the thermostability of single-domain antibodies (47, 48). This could lead to the development of OUD countermeasures that do not require special storage conditions allowing for their widespread implementation in diverse regions and populations.

## Experimental procedures

### Haptens

Fentanyl-based haptens F_1_, F_3_, and F_11_ and their conjugates used for isolation or characterization of anti-fentanyl VHH were obtained from Dr Pravetoni and previously described. The structure and synthesis of F_1_-biotin for BLI, F_3_-biotin for biopanning, and F_1_/F_3_/F_11_-BSA for ELISA were previously described (28, 29, 49).

### VHH identification

Using a previously described high diversity (>10^10^) camelid VHH phage display library a biopanning campaign against fentanyl and carfentanil haptens was carried out (26). Briefly, the library was incubated overnight with 1 μg of fentanyl hapten conjugated to biotin. The bound phage and biotinylated fentanyl hapten were then incubated at room temperature with magnetic Dynabeads, M-270 Streptavidin (Thermo Fisher Scientific) for 1 h. The resulting phage-opioid-bead complexes were washed with PBST and PBS before the remaining phage was eluted with 1 ml 100 mM triethylamine for 8 min before being neutralized with 500ul of 1M Tris-HCl, pH 7.5. Eluted phage was then used to infect TG1 *E. coli* for 1 h at 37C before being plated on 2xYT agar plates (containing Ampicillin and Glucose, AG) and grown overnight at 37 °C. The following day the infected TG1 growths were collected and resuspended in 36 ml of 2xYT-AG media. To generate more phage the TG1 growth was used to inoculate a new 100 ml culture and grown at 37 °C with shaking at 250 rpm until reaching an OD_600_ = 0.8. Once reaching the desired OD_600_ the phage-infected TG1 bacteria was co-infected with M13K07 helper phage and grown overnight at 30 °C with 250 rpm. The next day the media from the coinfected culture was harvested and the phage was purified with standard PEG precipitation methods (50). Following round three the eluted phage was also used to infect SS320 *E. coli* and plated in a dilution series to obtain single colony growths. The single colonies were then collected, grown, and induced with 1 mM isopropyl-β-D-thiogalactopyranoside. The supernatant media containing the induced camelid antibodies was evaluated using a previously described ELISA method to identify strong binders against the fentanyl hapten and an additional ELISA was performed to evaluate any cross-reactivity with a carfentanil hapten (26, 50, 51). Strong binding colonies were isolated and purified for further studies described below and sequenced to identify unique antibody leads.

### Generation of mammalian expression vectors

Fentanyl-binding VHH homodimer expression vectors consist of an open reading frame (ORF) that initiates with a murine IGHV signal peptide (MGWSCIILFLVATATGVHS), followed by two copies of the VHH encoding gene linked together with a (Gly_4_Ser)_4_ polypeptide linker and a c-terminal 6xHis tag for affinity purification. To facilitate expression vector assembly, codon optimized gene fragments encoding VHH homodimer were synthesized (Twist Bioscience) and cloned into pcDNA3.4 expression plasmids *via* Gibson assembly (21).

### Mammalian expression and purification

Logistically, recombinant expression of fentanyl-binding VHH homodimers were most easily produced *via* transient expression with the Expi293 expression system according to manufacturer instructions (ThermoFisher Catalog # A14635). Cell culture supernatant was harvested 7 days following transfection, and VHH homodimers were purified *via* liquid chromatography on an ÄKTA pure (Cytiva) with a HisTrap excel column (Cytiva Product # 29048586). The chromatographic process included column equilibration in 20 mM NaPO_4_, 0.5 M NaCl, pH 7.4 followed by sample loading and a wash step with 20 mM NaPO_4_, 0.5 M NaCl, 10 mM imidazole, pH 7.4 to remove non-specifically bound protein contaminants. His-tagged VHH homodimer eluted during the isocratic application of 20 mM NaPO_4_, 0.5 M NaCl, 500 mM imidazole, pH 7.4. Eluate fractions were pooled and buffer exchanged into PBS, pH 7.4. Protein concentration was determined by absorbance at 280 nm on a Nanodrop and purity analysis was performed by SDS-PAGE (Fig. S7). The maintenance of binding with mammalian-produced JGFN4 indicated that changing from bacterial to mammalian expression was acceptable. SDS-PAGE indicated the presence of N-linked glycosylation in the mammalian sample, but after removing glycosylation with PNGase, no change in binding was observed (data not shown).

### X-ray crystallographic studies

JGFN4 used in the structural studies was expressed with a C-terminal 6xHis-tag in SHuffle T7 Express *E. coli* strain (NEB) and purified over Ni-NTA Superflow (Qiagen) and Superdex 75 (Cytiva) chromatography columns. Discrete peaks corresponding to VHH monomers and dimers were obtained (Fig. S3). Each species was concentrated by ultrafiltration in 20 mM Tris-HCl, pH 7.4, 0.5 M NaCl, flash-frozen in liquid nitrogen, and stored at −80 °C. Protein concentrations were determined based on UV absorbance at 280 nm and the theoretical extinction coefficient. JGFN4 monomer or dimer at ∼12 mg ml^−1^ was mixed with a 5-fold molar excess of fentanyl and subjected to crystallization screening in the sitting drop vapor diffusion mode at ambient temperature, by mixing 0.1 μl each of the complex and reservoir solutions. Crystals used in the structural studies were obtained with the reservoir solutions of 0.1 M Tris-HCl, pH 8.0, 0.2 M LiCl, 20% (w/v) PEG 6000 for the crystal in space group *C*222_1_ (from dimeric JGFN4), and 0.2 M sodium acetate, 0.1 M sodium cacodylate pH 6.5, 30% (w/v) PEG 8000, for the crystal in space group P2_1_2_1_2 (from monomeric JGFN4), respectively. JGFN4 monomer without fentanyl was crystallized with the reservoir solution: 20% (w/v) PEG MME 2000, 0.1 M Tris-HCl pH 8.5, 0.2 M trimethylamine N-oxide. JGFN4 N76D was purified and subjected to crystallization screening as above. Crystals of monomeric JGFN4 N76D bound to fentanyl were obtained with the reservoir solution containing 0.1 M Tris-Bicine, pH 8.5, 0.02 M each of monosaccharides (D-glucose; D-mannose; D-galactose; L-fucose; D-xylose; N-acetyl-D-glucosamine), 20% (v/v) PEG 500 MME, 10% (w/v) PEG 20000. Crystals of dimeric JGFN4 N76D bound to fentanyl were obtained with the reservoir solution containing 0.2 M sodium acetate, 20% (w/v) PEG3350. The crystals were cryo-protected by a brief soaking in the respective reservoir solution supplemented with 25% ethylene glycol and flash-cooled by plunging in liquid nitrogen. X-ray diffraction data were collected at the Advanced Photon Source NE-CAT beamline 24-ID-C and 24-ID-E. All X-ray diffraction data were processed using XDS (52). Initial phases were obtained by molecular replacement with PHASER (53) using Gβγ VHH (PDB ID 6B20) (54) as the search model. Model building and refinement were done using COOT and PHENIX, respectively (55, 56). The summary of data collection and model refinement statistics is shown in Table 1. The atomic coordinates and structure factors have been deposited in the Protein Data Bank under the accession codes 8V9W, 8V9Y, 8V9X, 8V9Z, and 8VA0.

### Biolayer interferometry

Affinity of VHH homodimer was evaluated by biolayer interferometry (BLI) using an Octet R8 (Sartorius) with streptavidin-coated biosensors (Sartorius Catalog # 18–5020). F_1_-biotin was loaded onto biosensors at 0.2 μg/ml in phosphate-buffered saline with 0.1% bovine serum albumin (BSA) for 60 s. Association of VHH homodimer to immobilized hapten was measured over the course of 5 min at VHH homodimer concentrations of 100, 250, and 500 nM or 50, 100, and 200 nM, followed by dissociation in phosphate-buffered saline with 0.1% BSA for 5 min. All kinetic parameters including on-rate (k_on_), off-rate (k_off_), and K_D_ (k_off_/k_on_) were calculated using Octet software (Sartorius) were determined with a global curve fitting.

### Concentration ELISA

VHH concentration to obtain suitable signal for competitive ELISA against fentanyl hapten was determined by concentration ELISA. Briefly, high-binding polystyrene 96-well assay plates (Corning) were coated with 0.05 μg/ml F_1_-BSA in carbonate-bicarbonate buffer, pH 9.5 (Thermo) overnight, followed by washing and blocking with 1% fish gelatin (Sigma G7041) in PBS-T buffer (ThermoFisher 28,352) for 1.5 h. Plates were loaded with a serial dilution of VHH homodimer (0–0.5ug/ml) and incubated for 2 h. Plates were incubated with 1:300 diluted HRP-labeled anti-histidine tag secondary antibody (Santa Cruz Biotechnology Cat # sc-53073) overnight. HRP activity was quantitated with TMB Chromogen Solution (ThemoFisher 002,023) *via* absorbance measurement at 450 nm on a Victor Nivo plate reader (PerkinElmer). EC50 of VHH for fentanyl hapten was measured as the concentration of VHH needed to obtain 50% maximum TMB signal (EC_50_) (Fig. S8 and Table S4).

### Competitive ELISA

The relative affinity of VHH for fentanyl was determined by competitive ELISA. Briefly, high-binding polystyrene 96-well assay plates (Corning) were coated with 0.05 μg/ml F_1_-BSA in carbonate-bicarbonate buffer, pH 9.5 (Thermo) overnight, followed by washing and blocking with 1% fish gelatin (Sigma G7041) in PBS-T buffer (ThermoFisher 28,352) for 1.5 h. Plates were loaded with a serial dilution of free fentanyl in PBS-T (37 uM–70 nM), and VHH homodimer samples were applied (0.25ug/ml low-affinity VHH or 0.015ug/ml high-affinity VHH) and incubated for 2 h. Plates were incubated with HRP-labeled anti-histidine tag secondary antibody (Santa Cruz Biotechnology Cat # sc-53073) overnight. HRP activity was quantitated with TMB Chromogen Solution (ThemoFisher 002,023) *via* absorbance measurement at 450 nm on a Victor Nivo plate reader (PerkinElmer). The relative affinity of VHH for fentanyl was measured as the concentration of fentanyl reducing TMB signal by 50% (IC_50_).

### Differential scanning fluorimetry

The affinity of VHH was assessed using DSF to measure ligand-induced stabilization of protein unfolding with Protein Thermal Shift Dye Kit (ThermoFisher Catalog # 4461146). VHH homodimer (3 μM) was incubated in the presence of 0 to 1.5 mM fentanyl and subjected to a 25 to 95 °C thermal ramp at a rate of 0.015° C/sec using a QuantStudio 7 RT-PCR system (ThermoFisher). Fluorescence data were captured during the thermal ramp using the system’s X4/M4 optical filter settings. Melting temperature data was analyzed with Protein Thermal Shift Software v1.4 (ThermoFisher Catalog # 4466038), and Boltzmann T_m_ data for each sample were obtained. The affinity of VHH homodimers for fentanyl was calculated from the concentration-dependent increase in Boltzmann T_m_ according to the method described (57).

## Data availability

All critical data to support the findings in this article are included. Structural data are available at the PDB. Additional data will be made available by the corresponding authors upon request.

## Supporting information

This article contains supporting information.

## Conflict of interest

The authors declare the following financial interests/personal relationships which may be considered as potential competing interests: A. M. L, J. P. G, M. P., C. B., and D. H. are co-inventors on a provisional patent application USPTO--231220--09824.412.
