# Supplementary material for: Identification and biophysical characterization of a novel domain-swapped camelid antibody specific for fentanyl
Source: J Biol Chem. 2024 Jun 28;300(8):107502. doi: 10.1016/j.jbc.2024.107502 (PMC11321312; doi:10.1016/j.jbc.2024.107502)
Supplement: Supporting information [file mmc1.docx]

**SUPPORTING INFORMATION**

Identification and biophysical characterization of a novel domain-swapped camelid antibody specific for fentanyl

Joseph P. Gallant^a*^, Dustin Hicks^b*^, Ke Shi^c^, Nicholas H. Moeller^c^, Brooke Hoppe^d^, Eric W. Lake^a^, Carly Baehr^b‡^, Marco Pravetoni^d,e,f‡^, Hideki Aihara^d‡^ , Aaron M. LeBeau^a,f,g‡^

^a^Department of Pathology and Laboratory Medicine, University of Wisconsin School of Medicine and Public Health, Madison, WI 53705, USA

^b^Department of Pharmacology, University of Minnesota Medical School, Minneapolis, MN 55455, USA

^c^Department of Biochemistry, Molecular Biology, and Biophysics, University of Minnesota, Minneapolis, MN 55455, USA

^d^Department of Psychiatry and Behavioral Sciences, University of Washington School of Medicine, Seattle, WA USA

^e^Center for Medication Development for Substance Use Disorders, Seattle, WA, USA

^f^Department of Radiology, University of Wisconsin School of Medicine and Public Health, Madison, WI 53705, USA

^g^Carbone Cancer Center, University of Wisconsin-Madison, Madison, WI 53705, USA

**Supporting Material Contents:**

1. **Figure S1. Structures of opioid haptens used in this study.**
2. **Figure S2. DNA and amino acid sequences of JGFN1 – 5**
3. **Figure S3. JGFN4 nanobody exists as a mixed monomer and dimer in solution.**
4. **Figure S4.** **JGFN4 monomer does not bind to fentanyl hapten by BLI.**
5. **Figure S5. Side-by-side comparison of the structures of fentanyl-free JGFN4 WT monomer and JGFN4 WT bound to fentanyl as the homodimer**
6. **Figure S6. Cartoon of (G_4_S)_4_-linked JGFN4 dimer**
7. **Figure S7. Representative SDS-PAGE of mammalian expressed (G_4_S)_4_-linked JGFN4 dimer constructs**
8. **Figure S8. Concentration ELISA Data EC50 curves**
9. **Figure S9. Representative BLI curves.**
10. **Figure S10. Representative DSF Curves**
11. **Figure S11. DSF K_D_. ΔG (kJ) vs T (K) plots for each VHH.**
12. **Table S1. BLI K_D_ (M) data.**
13. **Table S2. BLI Ka (1/Ms) data.**
14. **Table S3. BLI Kdis (1/s) data.**
15. **Table S4. Concentration ELISA data.**
16. **Table S5. Competitive ELISA data.**
17. **Table S6. DSF K_D_ Data.**
18. **Table S7. Raw T_m_B data for S29Y/N76D mutant KD analysis**

**Figure S1.** **Structures of opioid haptens used in this study.**

**F3 hapten, unconjugated.**


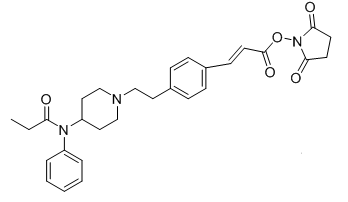


**Biotinylated F1 hapten used for BLI**


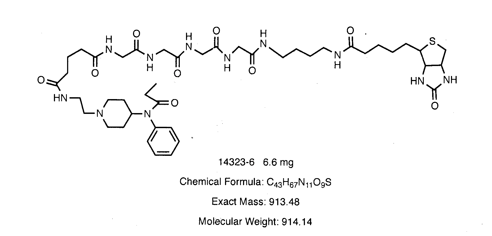


**
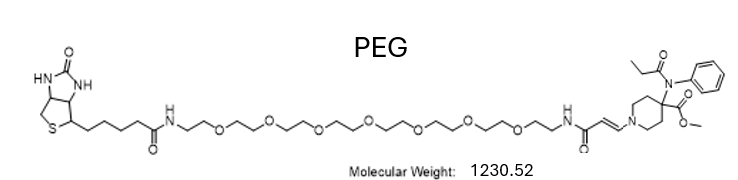
F11 biotinylated hapten with PEG linker**

**Figure S2. DNA and amino acid sequences of JGFN1 - 5**

**JGFN1**

DNA:ATGAAATACCTATTGCCTACGGCGGCCGCTGGATTGTTATTACTCGCGGCCCAGCCGGCCATGGCTCAGGTGCAGCTCGTGGAGTCTGGGGGAGGCTTGGTGCAGGCTGGAGAGTCTCTGAAACTCTCCTGTAAGGCCTCTGGAGATACCTTCGGTATCACCGTCATCGGCTGGCACCGCCAACGTGCAGGTCAACAGCAGCGCGAATTGGTCGCGCGCATTTATAATACTGGGACTGTAAAATACGCCGACTCCGTGAAGGGCCGGTTCACCCTCTCCAGTGACAGCGCCAACGACGTGGTGTATCTGGAGATGACTGACTTGAAACCGGAAGACACGGCCGTCTATTACTGCCACGCGATGGGCGAAGCTGACCTCACTCACTATGACCTCTGGGGCCCGGGGACCCAGGTCACCGTCTCCAGCGGCCCGGGAGGCCAACACCATCACCACCATCATGGCGCATATCCGTATGATGTGCCGGACTATGCTTCTTAG

AA:MKYLLPTAAAGLLLLAAQPAMAQVQLVESGGGLVQAGESLKLSCKASGDTFGITVIGWHRQRAGQQQRELVARIYNTGTVKYADSVKGRFTLSSDSANDVVYLEMTDLKPEDTAVYYCHAMGEADLTHYDLWGPGTQVTVSSGPGGQHHHHHHGAYPYDVPDYAS*

**JGFN2**

DNA:ATGAAATACCTATTGCCTACGGCGGCCGCTGGATTGTTATTACTCGCGGCCCAGCCGGCCATGGCTCAGGTGCAGCTCGTGCAGTCTGGGGGAGGCTTGGTGCAACCTGGGGGTTCTCTGAGACTCTCCTGTGCAGCCTCTGGCTTCACCCTCAGTACGTTGGATATGAGGTGGTTCCGCCAGGCTCCAGGAAAGGGGTTCGAGTGGGTCTCAACTGTTAGTCCTGATGGTAACACGTACTACTCAGACTCCGCGAAGGGCCGATTCGCCATCTCCAGAGACGTCGCCAAGAACACGGTGAATCTGCAAATGAGCAGCCTGAAACCTGAAGACACGGCCGTCTATTATTGTAATGCAGATATTTCAATCGCGGCGCACGAGGGCGGACTGCCCTACTGGGGCAAAGGGACCCCGGTCACCGTCTCCAGCGGCCCGGGAGGCCAACACCATCACCACCATCATGGCGCATATCCGTATGATGTGCCGGACTATGCTTCTTAG

AA:MKYLLPTAAAGLLLLAAQPAMAQVQLVQSGGGLVQPGGSLRLSCAASGFTLSTLDMRWFRQAPGKGFEWVSTVSPDGNTYYSDSAKGRFAISRDVAKNTVNLQMSSLKPEDTAVYYCNADISIAAHEGGLPYWGKGTPVTVSSGPGGQHHHHHHGAYPYDVPDYAS*

**JGFN3**

DNA:ATGAAATACCTATTGCCTACGGCGGCCGCTGGATTGTTATTACTCGCGGCCCAGCCGGCCATGGCTGATGTTCAGCTCGTGGAGTCTGGGGGAGGCATGGTGCAGCCTGGGGGGTCTCTGAGACTCTCCTGTGTAACCTCTGGATTCGACTTCAGCCGCTATGATATGGGCTGGGTCCGCCAGGCTCCAGGGAAGGGGCCCGAGTGGGTCTCAGGAATTAAAAGTGGTGGCGGTCCCACAGTCTACCTAGACTCCGTGAAGGGCCGATTCACCGTCTCCAGAGATAACGCCAAGAATACTTTATATCTCCAAATGAACAACCTGAAACCTGAGGACACGGCCCGGTATTACTGTGCGAGAGATCCCGACGACCACAGTGGTAGTTACTGGGGCGACTACTGGGGCCAGGGGACCCTGGTCACCGTCTCCAGCGGCCCGGGAGGCCAACACCATCACCACCATCATGGCGCATATCCGTATGATGTGCCGGACTATGCTTCTTAG

AA:MKYLLPTAAAGLLLLAAQPAMADVQLVESGGGMVQPGGSLRLSCVTSGFDFSRYDMGWVRQAPGKGPEWVSGIKSGGGPTVYLDSVKGRFTVSRDNAKNTLYLQMNNLKPEDTARYYCARDPDDHSGSYWGDYWGQGTLVTVSSGPGGQHHHHHHGAYPYDVPDYAS*

**JGFN4**

DNA:ATGAAATACCTATTGCCTACGGCGGCCGCTGGATTGTTATTACTCGCGGCCCAGCCGGCCATGGCTCAGGTGCAGCTCGTGGAGTCCGGGGGAGGCTTGGCGCAGGCTGGGGGATCTCTGCAACTCTCGTGCGCAGCTTCCGGTAGTACCTCCAGAGTCAATGCCATGGGTTGGTACCGCCAGACTCCGGGGAAAGAGCGCGAGTTGGTCGCGGCGATTGACCGCTCCGGAGCCACGGTCTACTCGGAGTCCGTGAGGGGACGATTCACCATCTCCAAAAACGATGCCAAAAACATCGTGTGGCTGCAAATGAACAACCTGACGACTGAAGATACGGCCGTCTATTACTGTCGCAGTGGGGTCCTTGGTTCCTGGGGCCTGGGGACCCAGGTCACCGTCTCCAGCGGCCCGGGAGGCCAACACCATCACCACCATCATGGCGCATATCCGTATGATGTGCCGGACTATGCTTCTTAG

AA:MKYLLPTAAAGLLLLAAQPAMAQVQLVESGGGLAQAGGSLQLSCAASGSTSRVNAMGWYRQTPGKERELVAAIDRSGATVYSESVRGRFTISKNDAKNIVWLQMNNLTTEDTAVYYCRSGVLGSWGLGTQVTVSSGPGGQHHHHHHGAYPYDVPDYAS*

**JGFN5**

DNA:ATGAAATACCTATTGCCTACGGCGGCCGCTGGATTGTTATTACTCGCGGCCCAGCCGGCCATGGCTCAGGTGCAGCTCGTGGAGTCTGGGGGAGGCTTGGTGCAGGCTGGAGAATCTCTGAATCTCTCCTGCACAGCCTCCGGGAACACCTTCAGTATCAATTCCATGGCCTGGTACCGCCAGGCTCCAGGAAAGCAGCGCGAGTTGGTCGCGGAACTTCATAAAACTGATCGGCCAGGTAGTGTCGCCGCGAACTATGCAGACTCCGTGAAGGGCCGATTCACCATCGCCACCGACAACTCCAGAGACACGATGTATCTCCAAATGACCAACCTAAAATCTGCGGATACTGCCACCTATTTCTGTTATGCGACGGGGCTCTGGAGGGATCATGAAGTTTGGGGCCAGGGCACCCTGGTCACCGTCTCCAGCGGCCCGGGAGGCCAACACCATCACCACCATCATGGCGCATATCCGTATGATGTGCCGGACTATGCTTCTTAG

AA:MKYLLPTAAAGLLLLAAQPAMAQVQLVESGGGLVQAGESLNLSCTASGNTFSINSMAWYRQAPGKQRELVAELHKTDRPGSVAANYADSVKGRFTIATDNSRDTMYLQMTNLKSADTATYFCYATGLWRDHEVWGQGTLVTVSSGPGGQHHHHHHGAYPYDVPDYAS*

PelB leader sequence in yellow

6x his tag in blue

HA tag in green

VHH sequence in pink

CDRs in teal

**JGFN4 WT Homodimer AA Sequence:**

MGWSCIILFLVATATGVHSQVQLVESGGGLAQAGGSLQLSCAASGSTSRVNAMGWYRQTPGKERELVAAIDRSGATVYSESVRGRFTISKNDAKNIVWLQMNNLTTEDTAVYYCRSGVLGSWGLGTQVTVSSGPGGQGGGGSGGGGSGGGGSGGGGSQVQLVESGGGLAQAGGSLQLSCAASGSTSRVNAMGWYRQTPGKERELVAAIDRSGATVYSESVRGRFTISKNDAKNIVWLQMNNLTTEDTAVYYCRSGVLGSWGLGTQVTVSSGPGGQHHHHHH*

mIGHV leader sequence in yellow

VHH sequence in pink

Linker in green

**Figure S3. JGFN4 nanobody exists as a mixed monomer and dimer in solution.**


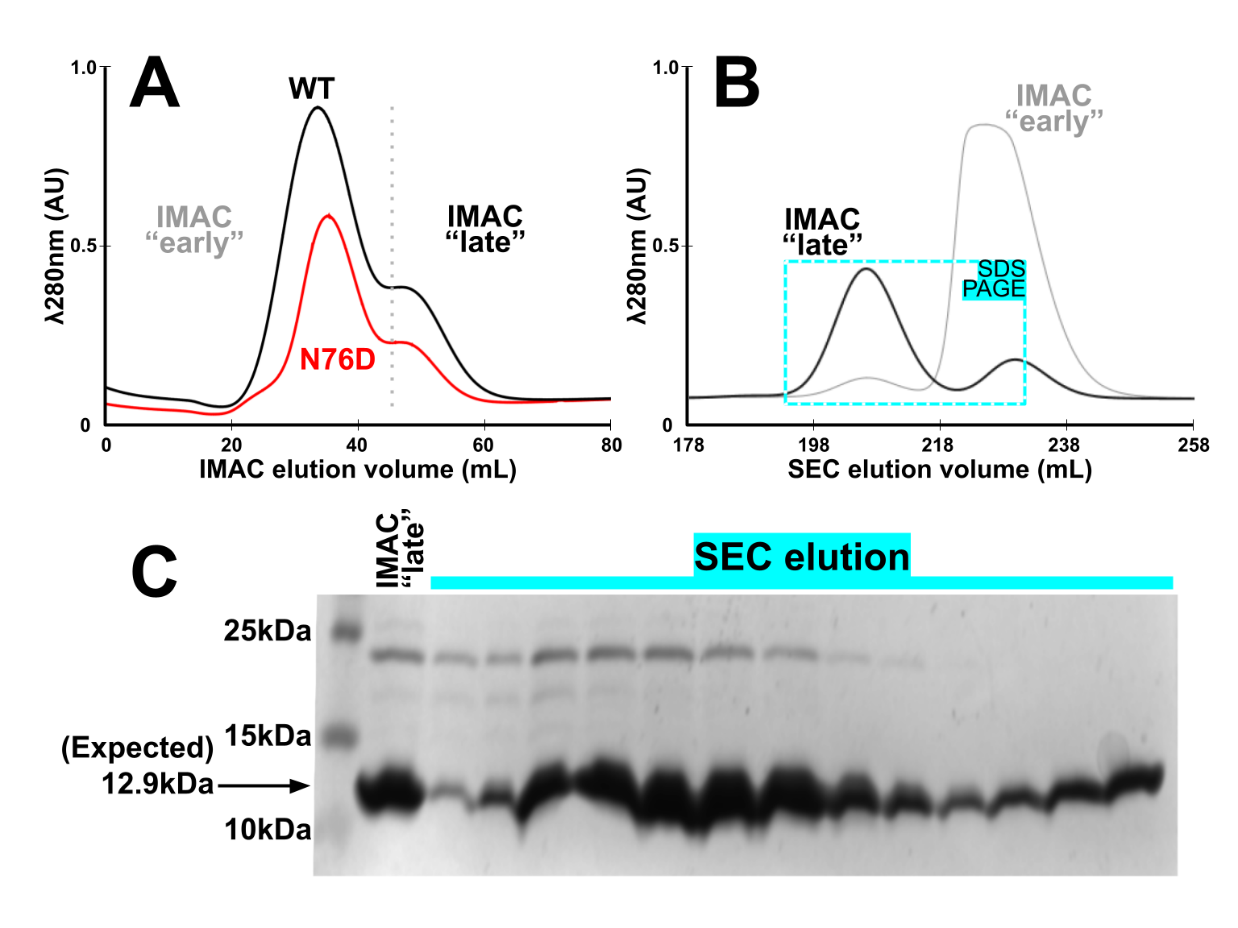


(A) Ni^2+^ immobilized metal affinity chromatography (IMAC) elution profiles for the wild type (black) and N76D mutant (red) JGFN4 expressed in *E. coli*. The proteins were eluted with a linear concentration gradient of imidazole. Two distinct elution peaks were observed and separated into ‘early’ and ‘late’ pools, as shown by the dashed line. (B) Separate analyses of the “early” and “late” IMAC pools by size exclusion chromatography (SEC) reveal two distinct peaks. (C) Denaturing gel electrophoresis of the “IMAC late” SEC elution fractions (boxed in B) shows that both peaks are largely comprised of a 12.9 kDa protein, expected for the JGFN4 nanobody. The species that elutes early in IMAC and late in SEC is likely to be a monomer of JGFN4, whereas the species that elutes late in IMAC and early in SEC is likely to be a dimer of JGFN4. The results also suggest that the monomer and dimer do not equilibrate rapidly.

**Figure S4.** **JGFN4 monomer does not bind to fentanyl hapten by BLI. BLI binding chromatogram of “early” (labeled as Ni Peak 1) and “late” (labeled as Ni Peak 2) IMAC pools. “Early” peak corresponding to dimer binds to fentanyl hapten, however “late” peak corresponding to monomer does not.**

**
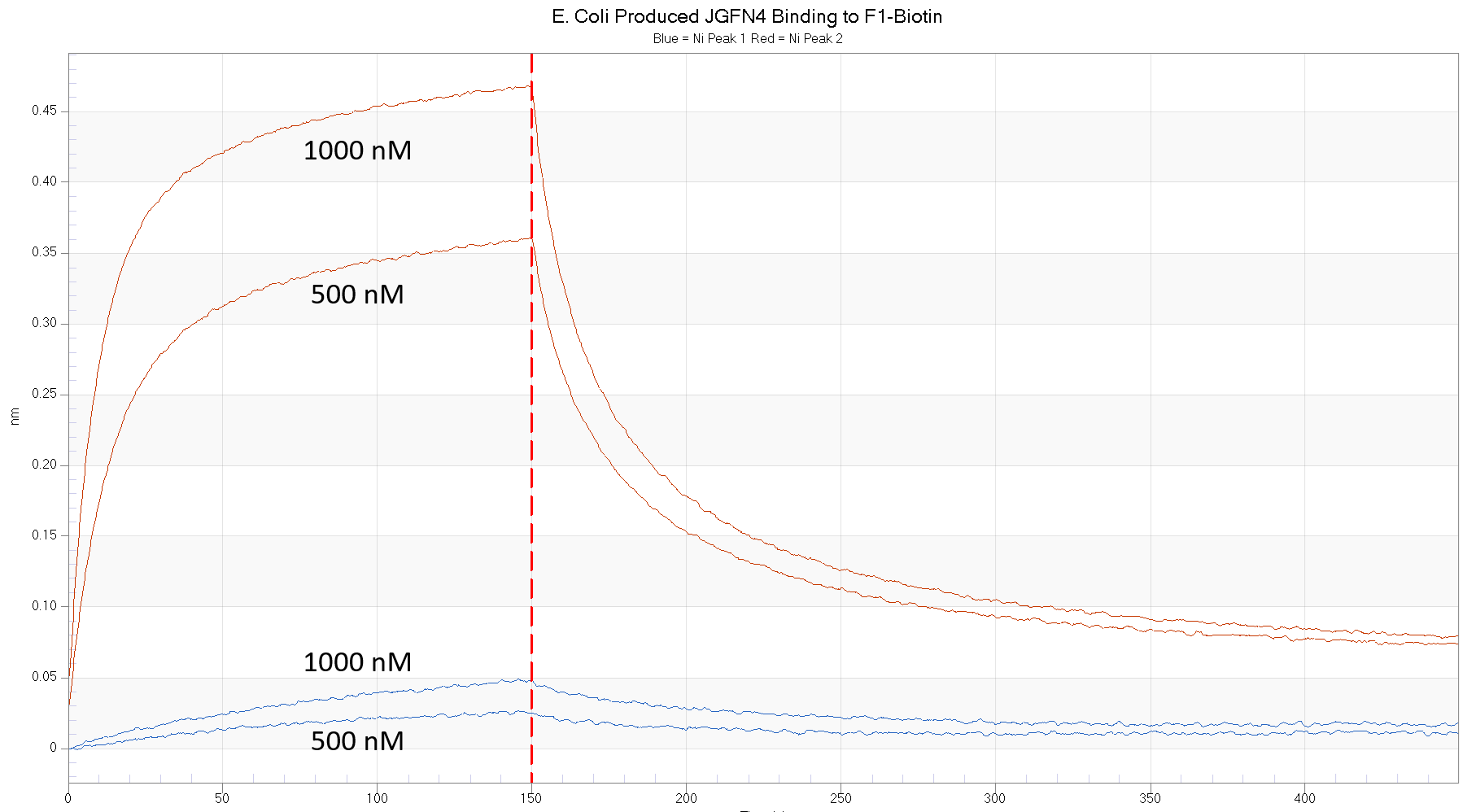
**

**Figure S5. Side-by-side comparison of the structures of fentanyl-free JGFN4 WT monomer and JGFN4 WT bound to fentanyl as the homodimer**


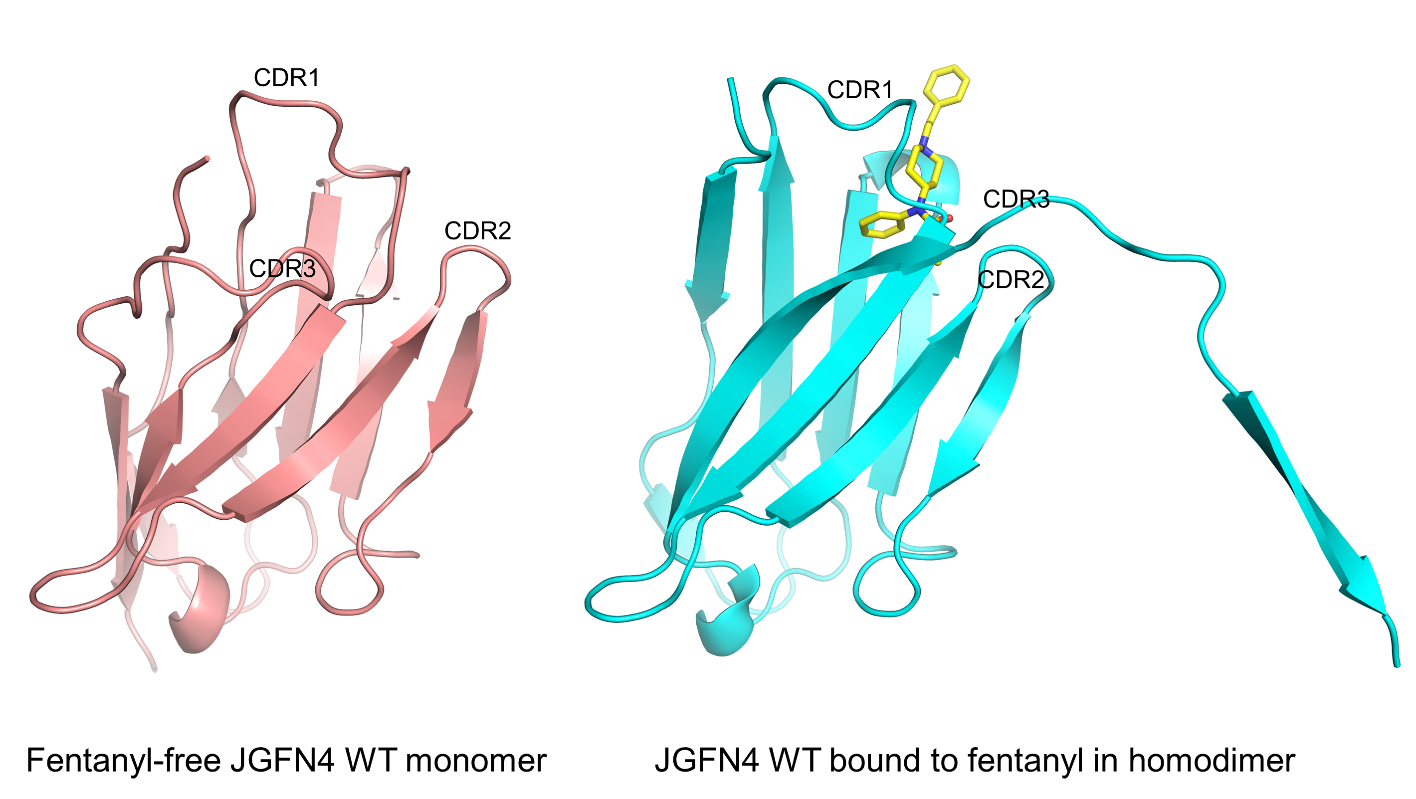


**Figure S6. Cartoon of (G_4_S)_4_-linked JGFN4 dimer**


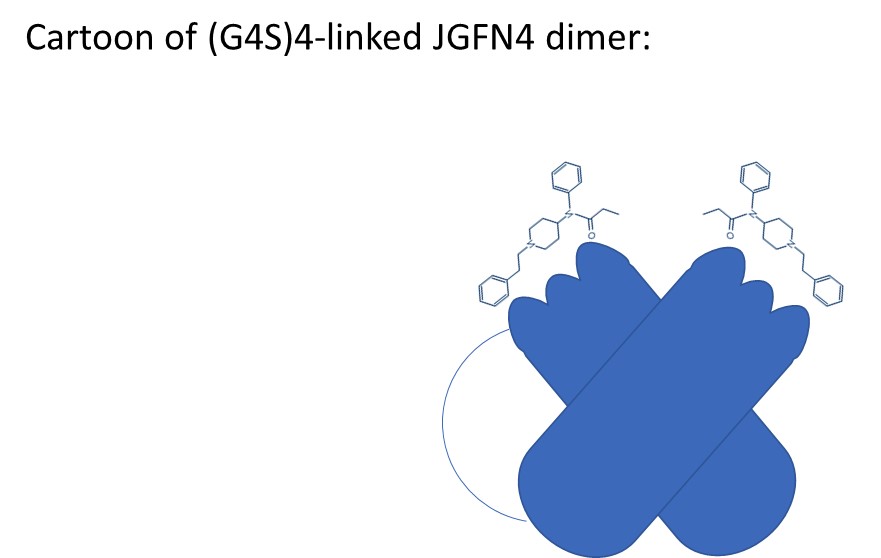


**Figure S7. Representative SDS-PAGE of mammalian expressed (G_4_S)_4_-linked JGFN4 dimer constructs**


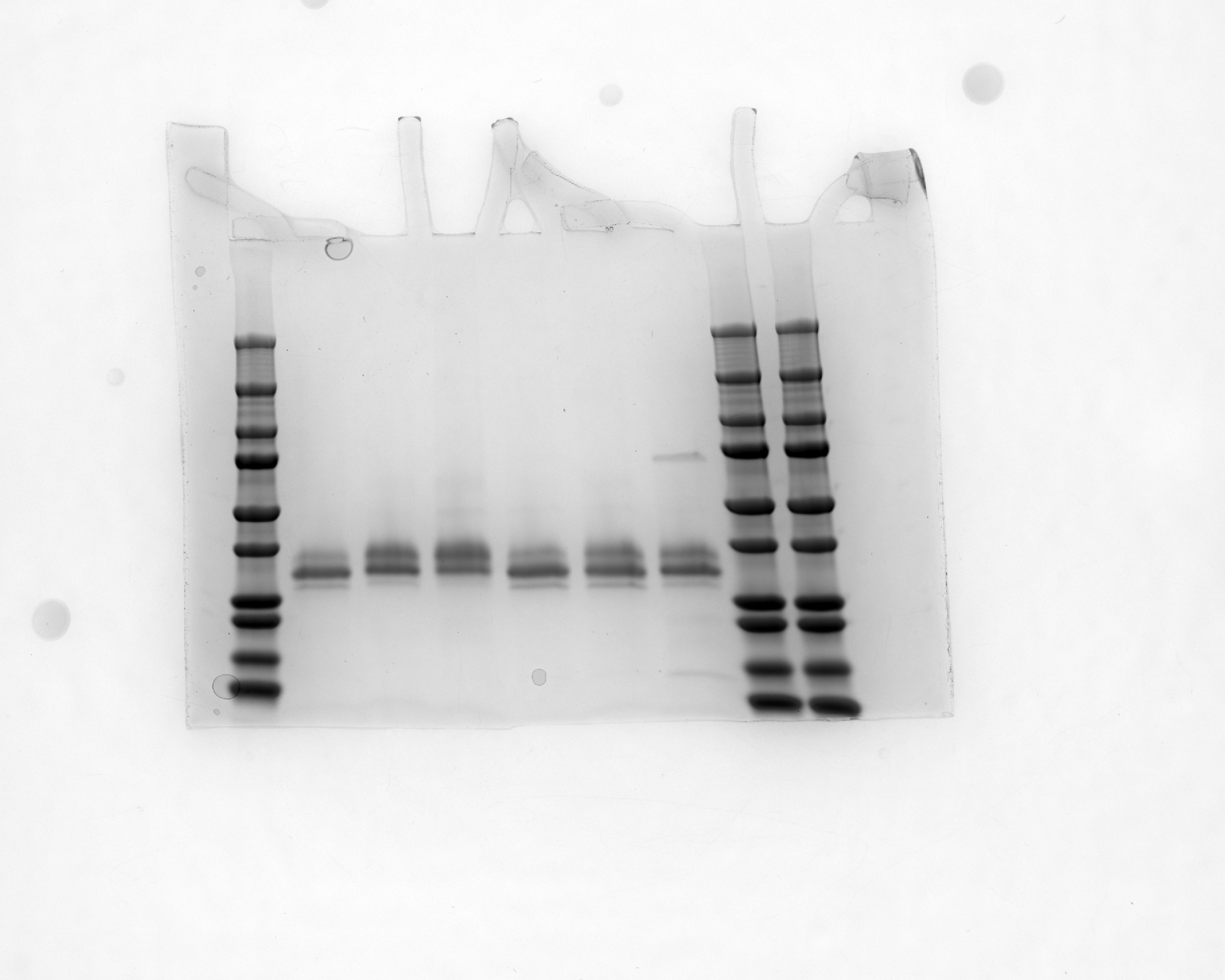


WT

50 kDa

25 kDa

15 kDa

10 kDa

20 kDa

37 kDa

A74Y

A74W

R53H

N76D

N76H

**Table S1. BLI K_D_ (M) data. Average of two replicate curves, generated from the testing of each VHH at 3 concentrations.**

| Mutations | Average KD (M) | Standard Deviation | SEM | KD Replicate 1 (M) | KD Replicate 2 (M) |  |
| --- | --- | --- | --- | --- | --- | --- |
| WT | **1.07E-08** | **8.10E-10** | **5.73E-10** | 1.01E-08 | 1.13E-08 |  |
| A74Y | **1.20E-08** | **6.51E-11** | **4.61E-11** | 1.19E-08 | 1.20E-08 |  |
| A74W | **1.63E-08** | **1.13E-09** | **7.98E-10** | 1.71E-08 | 1.55E-08 |  |
| *R53H* | *1.64E-09* |  |  | *1.64E-09* |  | *questionable/weak binding* |
| N76D | **8.46E-09** | **6.54E-10** | **4.62E-10** | 8.92E-09 | 7.99E-09 |  |
| N76H | **1.36E-08** | **1.46E-10** | **1.03E-10** | 1.35E-08 | 1.37E-08 |  |
| A74Y/N76D | **5.80E-09** | **2.05E-09** | **1.45E-09** | 4.35E-09 | 7.25E-09 |  |
| S29Y/N76D | **5.45E-09** | **4.38E-10** | **3.10E-10** | 5.14E-09 | 5.76E-09 |  |
| S29Y/A74Y/N76D | **5.43E-09** | **5.01E-10** | **3.54E-10** | 5.07E-09 | 5.78E-09 |  |
| *M34A* | *1.10E-11* |  |  | *1.10E-11* |  | *questionable/no binding* |
| *K71A* | *1.30E-11* |  |  | *1.30E-11* |  | *questionable/no binding* |
| *N32A* | *1.23E-11* |  |  | *1.23E-11* |  | *questionable/no binding* |

**Table S2. BLI Ka (1/Ms) data.**

| Mutations | Average Ka (1/Ms) | Standard Deviation | SEM | Ka Replicate 1 (1/Ms) | Ka Replicate 2 (1/Ms) |  |
| --- | --- | --- | --- | --- | --- | --- |
| WT | **1.27E+05** | **7.14E+03** | **5.05E+03** | 1.32E+05 | 1.22E+05 |  |
| A74Y | **2.59E+05** | **9.62E+03** | **6.80E+03** | 2.52E+05 | 2.65E+05 |  |
| A74W | **2.58E+05** | **2.22E+04** | **1.57E+04** | 2.42E+05 | 2.74E+05 |  |
| *R53H* | *8.40E+04* |  |  | *8.40E+04* |  | *questionable/weak binding* |
| N76D | **7.76E+04** | **4.17E+02** | **2.95E+02** | 7.79E+04 | 7.73E+04 |  |
| N76H | **1.70E+05** | **1.15E+04** | **8.15E+03** | 1.78E+05 | 1.62E+05 |  |
| A74Y/N76D | **6.66E+04** | **3.73E+03** | **2.64E+03** | 6.40E+04 | 6.93E+04 |  |
| S29Y/N76D | **6.42E+04** | **2.76E+03** | **1.96E+03** | 6.62E+04 | 6.23E+04 |  |
| S29Y/A74Y/N76D | **7.91E+04** | **5.18E+03** | **3.67E+03** | 8.28E+04 | 7.54E+04 |  |
| *M34A* | *2.81E+04* |  |  | *2.81E+04* |  | *questionable/no binding* |
| *K71A* | *3.76E+04* |  |  | *3.76E+04* |  | *questionable/no binding* |
| *N32A* | *3.98E+04* |  |  | *3.98E+04* |  | *questionable/no binding* |

**Table S3. BLI Kdis (1/s) data.**

| Mutations | Average Kdis (1/s) | Standard Deviation | SEM | Kdis Replicate 1 (1/s) | Kdis Replicate 2 (1/s) |  |
| --- | --- | --- | --- | --- | --- | --- |
| WT | **1.36E-03** | **2.55E-05** | **1.80E-05** | 1.34E-03 | 1.37E-03 |  |
| A74Y | **3.10E-03** | **1.33E-04** | **9.40E-05** | 3.00E-03 | 3.19E-03 |  |
| A74W | **4.19E-03** | **7.14E-05** | **5.05E-05** | 4.14E-03 | 4.24E-03 |  |
| *R53H* | *1.38E-04* |  |  | *1.38E-04* |  | *questionable/weak binding* |
| N76D | **6.57E-04** | **5.42E-05** | **3.84E-05** | 6.95E-04 | 6.18E-04 |  |
| N76H | **2.31E-03** | **1.31E-04** | **9.25E-05** | 2.41E-03 | 2.22E-03 |  |
| A74Y/N76D | **3.90E-04** | **1.58E-04** | **1.12E-04** | 2.79E-04 | 5.02E-04 |  |
| S29Y/N76D | **3.49E-04** | **1.31E-05** | **9.25E-06** | 3.40E-04 | 3.59E-04 |  |
| S29Y/A74Y/N76D | **4.28E-04** | **1.15E-05** | **8.10E-06** | 4.20E-04 | 4.36E-04 |  |
| *M34A* | *3.09E-07* |  |  | *3.09E-07* |  | *questionable/no binding* |
| *K71A* | *4.88E-07* |  |  | *4.88E-07* |  | *questionable/no binding* |
| *N32A* | *4.88E-07* |  |  | *4.88E-07* |  | *questionable/no binding* |

**Table S4. Concentration ELISA data. Data used to select VHH concentration to use for optimal signal in competitive ELISA.**

| Mutation(s) | EC50 (M) | SD | SEM | IC50 replicate 1 | IC50 replicate 2 |
| --- | --- | --- | --- | --- | --- |
| WT | 0.0608 | 0.0056 | 0.0040 | 0.05681 | 0.06477 |
| A74Y | 0.0459 | 0.0060 | 0.0043 | 0.0416 | 0.05013 |
| A74W | 0.0313 | 0.0050 | 0.0035 | 0.02772 | 0.03481 |
| R53H | 0.2020 | 0.0801 | 0.0566 | 0.1453 | 0.2586 |
| N76D | 0.0135 | 0.0002 | 0.0001 | 0.01367 | 0.01341 |
| N76H | 0.0133 | 0.0010 | 0.0007 | 0.01257 | 0.01394 |
| A74Y/N76D | 0.0203 | 0.0012 | 0.0008 | 0.02113 | 0.0195 |
| S29Y/N76D | 0.0177 | 0.0002 | 0.0001 | 0.01756 | 0.01781 |
| S29Y/A74Y/N76D | 0.0096 | 0.0011 | 0.0008 | 0.008853 | 0.0104 |
| M34A | 1.4820 |  |  | Unstable | 1.482 |
| K71A | 0.2119 |  |  | Unstable | 0.2119 |
| N32A | No binding detected |  |  | Unstable | Unstable |

**Table S5. Competitive ELISA data.**

| Mutation(s) | VHH assay concentration (ug/mL) | IC50 (M) | SD | SEM | IC50 replicate 1 | IC50 replicate 2 | IC50 replicate 3 | IC50 replicate 4 |
| --- | --- | --- | --- | --- | --- | --- | --- | --- |
| WT | 0.25 | 2.26E-05 | 1.02E-05 | 5.11E-06 | 3.76E-05 | 1.51E-05 | 2.04E-05 | 1.72E-05 |
| A74Y | 0.25 | 2.48E-05 | 5.85E-06 | 3.38E-06 | 2.44E-05 | 1.91E-05 | 0.2257 | 3.08E-05 |
| A74W | 0.25 | 3.61E-05 | 2.27E-05 | 1.14E-05 | 1.72E-05 | 6.90E-05 | 3.17E-05 | 2.66E-05 |
| R53H | 0.25 | 1.78E-05 | 4.99E-06 | 2.49E-06 | 1.30E-05 | 1.42E-05 | 2.04E-05 | 2.35E-05 |
| N76D | 0.015 | 4.43E-06 | 3.56E-07 | 1.78E-07 | 4.14E-06 | 4.63E-06 | 4.83E-06 | 4.11E-06 |
| N76H | 0.015 | 7.95E-06 | 8.61E-07 | 4.30E-07 | 7.53E-06 | 7.67E-06 | 7.38E-06 | 9.23E-06 |
| A74Y/N76D | 0.015 | 4.14E-06 | 7.81E-07 | 3.90E-07 | 4.11E-06 | 5.24E-06 | 3.76E-06 | 3.45E-06 |
| S29Y/N76D | 0.015 | 5.35E-06 | 1.09E-06 | 5.46E-07 | 6.72E-06 | 4.66E-06 | 4.31E-06 | 5.70E-06 |
| S29Y/A74Y/N76D | 0.015 | 9.58E-06 | 4.81E-06 | 2.40E-06 | 1.08E-05 | 5.68E-06 | 5.98E-06 | 1.59E-05 |
| M34A |  | Not tested |  |  |  |  |  |  |
| K71A |  | Not tested |  |  |  |  |  |  |
| N32A |  | Not tested |  |  |  |  |  |  |

**Table S6. DSF K_D_ Data.**

| Mutation(s) | Kd (uM) determined at 600uM fentanyl | SD | SEM | KD replicate 1 | KD replicate 2 | KD replicate 3 |
| --- | --- | --- | --- | --- | --- | --- |
| WT | 1.10E-03 | 1.54E-04 | 8.91E-05 | 1.08E-03 | 1.27E-03 | 9.62E-04 |
| A74Y | 4.35E-04 | 5.22E-05 | 3.01E-05 | 4.93E-04 | 3.92E-04 | 4.21E-04 |
| A74W | 3.23E-04 | 1.62E-05 | 9.33E-06 | 3.05E-04 | 3.37E-04 | 3.25E-04 |
| R53H | 8.67E-04 | 2.67E-04 | 1.54E-04 | 8.78E-04 | 1.13E-03 | 5.94E-04 |
| N76D | 8.65E-05 | 1.79E-06 | 1.03E-06 | 8.76E-05 | 8.76E-05 | 8.45E-05 |
| N76H | 8.01E-05 | 5.67E-06 | 3.27E-06 | 7.67E-05 | 7.70E-05 | 8.66E-05 |
| A74Y/N76D | 4.26E-05 | 2.67E-06 | 1.54E-06 | 4.56E-05 | 4.20E-05 | 4.04E-05 |
| S29Y/N76D | 6.46E-06 | 3.50E-07 | 2.00E-07 | 6.60E-06 | 6.06E-06 | 6.72E-06 |
| S29Y/A74Y/N76D | 5.60E-07 | 7.00E-08 | 4.00E-08 | 5.73E-07 | 4.85E-07 | 6.30E-07 |
| M34A | 8.74E-04 | 2.41E-05 | 1.39E-05 | 9.01E-04 | 8.54E-04 | 8.68E-04 |
| K71A | 7.36E-04 | 1.66E-02 | 9.60E-03 | 5.27E-03 | -1.77E-02 | 1.46E-02 |
| N32A | 3.80E-02 | 6.45E-02 | 3.73E-02 | -5.41E-03 | 1.12E-01 | 7.19E-03 |

**Figure S8. Concentration ELISA Data EC50 curves. Concentration used in Competitive ELISA denoted by dotted line. Low signal of M34A, K71A, and N32A indicated lack of binding and exclusion from competitive ELISA.**

**Figure S9. Representative BLI curves.**

**A**


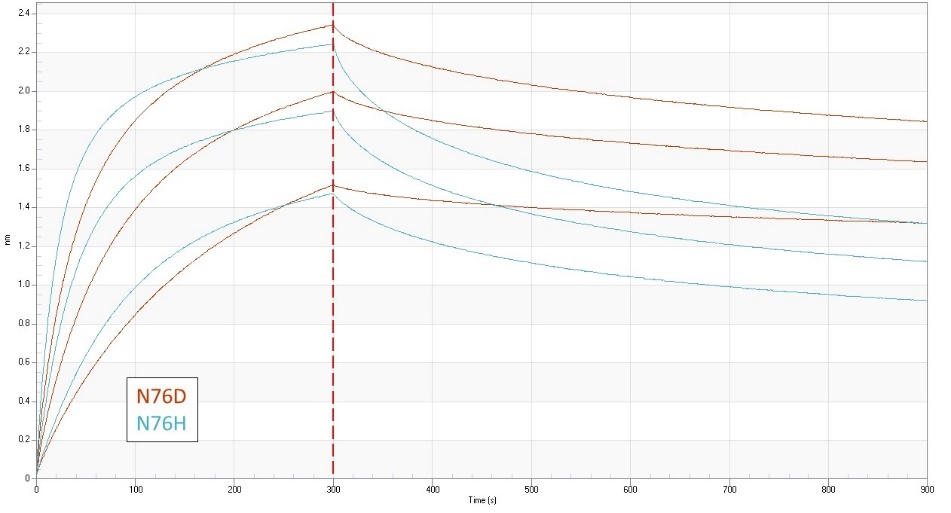


**B**

**
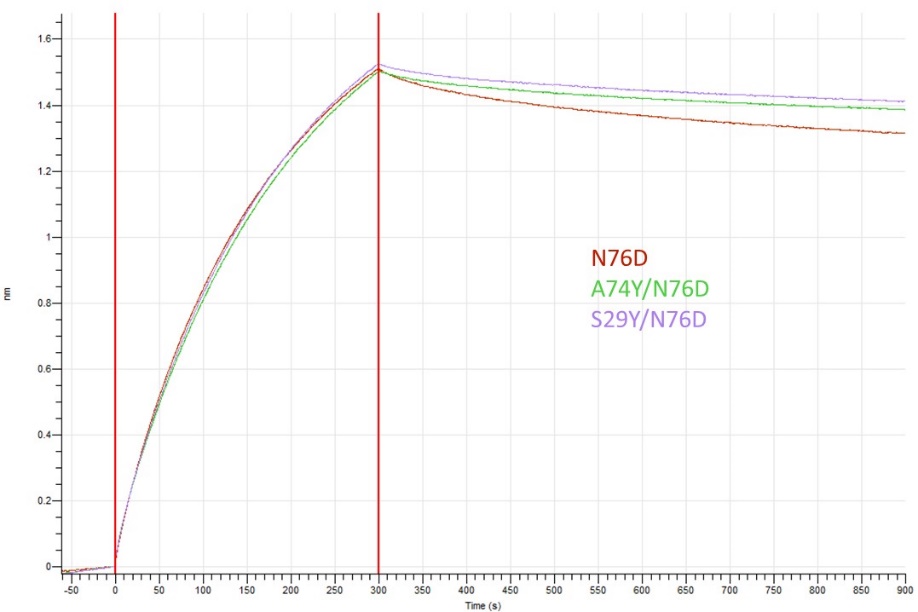
**

**C**


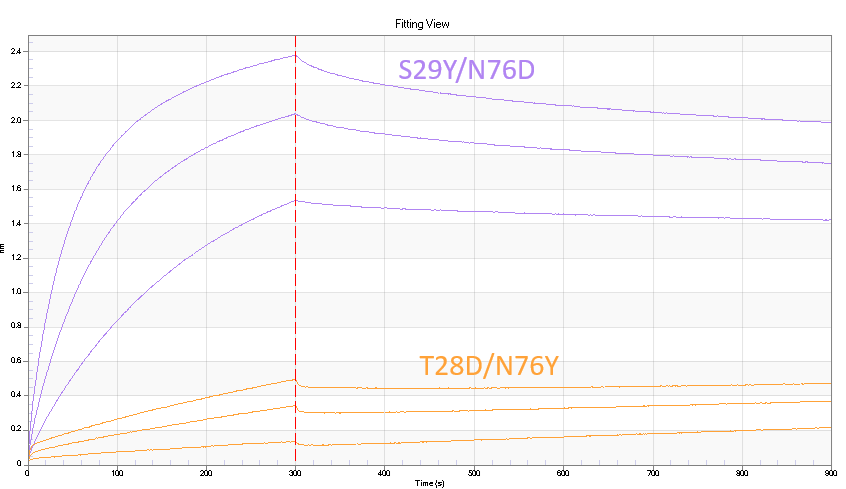


1. BLI curves for JGFN4 N76D and JGFN4 N76H at 3 concentrations (100, 200, 500 nM)
2. BLI curves of JGFN4 N76D, JGFN4 A74Y/N76D, and JGFN4 S29Y/N76D, all showing slightly decreased off rates but similar on rates for the successive mutants. Data is shown at a single concentration for clarity
3. BLI curves for JGFN4 S29Y/N76D and JGFN4 T28D/N76Y at 3 concentrations (100, 200, 500 nM) to show the loss in binding of the JGN4 T28D/N76Y mutant.

**Table S7. Raw T_m_B data for S29Y/N76D mutant KD analysis**

| Fentanyl Concentration (μM) | T_m_B (°C) | Average T_m_B (°C) |
| --- | --- | --- |
| 0 | 57.6 | 57.5 |
| 0 | 57.5 |  |
| 0 | 57.4 |  |
| 10 | 57.9 | 57.9 |
| 10 | 57.9 |  |
| 10 | 57.9 |  |
| 100 | 60.1 | 60.1 |
| 100 | 60 |  |
| 100 | 60.1 |  |
| 300 | 62.5 | 62.5 |
| 300 | 62.6 |  |
| 300 | 62.5 |  |
| 600 | 64.2 | 64.2 |
| 600 | 64.1 |  |
| 600 | 64.2 |  |
| 1000 | 65.6 | 65.5 |
| 1000 | 65.5 |  |
| 1000 | 65.5 |  |
| 1500 | 66.2 | 66.3 |
| 1500 | 66.4 |  |
| 1500 | 66.2 |  |

**Figure S10. Representative DSF Curves**


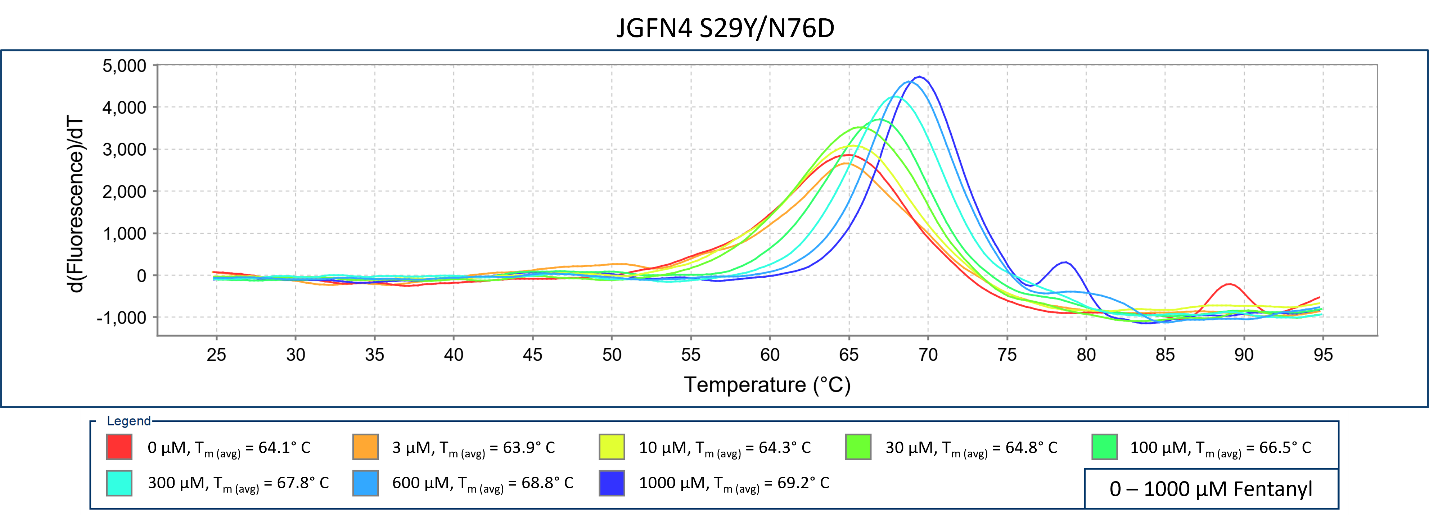

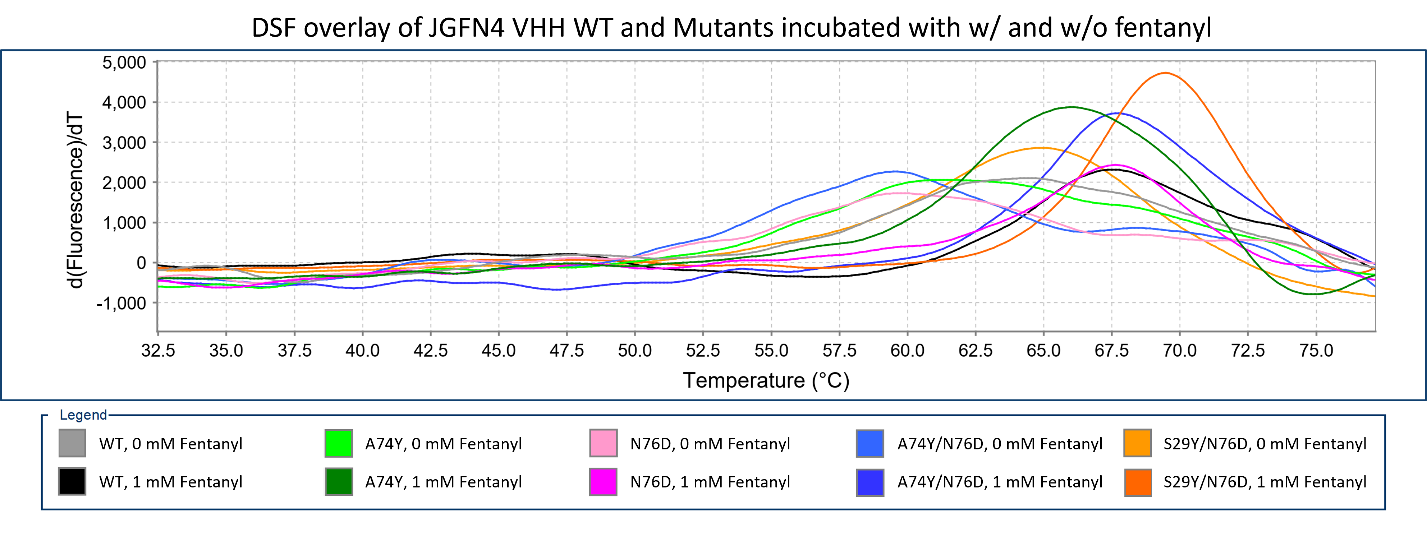


**Figure S11. DSF K_D_. ΔG (kJ) vs T (K) plots for each VHH.**
